# Measuring spatial inequalities in maternal and child mortalities in Pakistan: evidence from geographically weighted regression

**DOI:** 10.1186/s12889-024-19682-5

**Published:** 2024-08-16

**Authors:** Farzana Sher Muhammad, Sharifah Muhairah Shahabudin, Muzalwana Binti Abdul Talib

**Affiliations:** 1 Faculty of Business and Economics, Universiti Malaya, Kuala Lumpur, 50603 Malaysia; 2 Department of Economics, Faculty of Management Science, Sardar Bahadur Khan Womes University Balochistan, Quetta, 08763 Pakistan

**Keywords:** Maternal mortality, Child mortality, Disparity ratio, Univariate autocorrelation, Spatial heterogeneity, Geographically weighted regression, Pakistan

## Abstract

**Background:**

In developing countries, the death probability of a child and mother is more significant than in developed countries; these inequalities in health outcomes are unfair. The present study encompasses a spatial analysis of maternal and child mortalities in Pakistan. The study aims to estimate the District Mortality Index (DMI), measure the inequality ratio and slope, and ascertain the spatial impact of numerous factors on DMI scores across Pakistani districts.

**Method:**

This study used micro-level household datasets from multiple indicator cluster surveys (MICS) to estimate the DMI. To find out how different the DMI scores were, the inequality ratio and slope were used. This study further utilized spatial autocorrelation tests to determine the magnitude and location of the spatial dependence of the clusters with high and low mortality rates. The Geographically Weighted Regression (GWR) model was also applied to examine the spatial impact of socioeconomic, environmental, health, and housing attributes on DMI.

**Results:**

The inequality ratio for DMI showed that the upper decile districts are 16 times more prone to mortalities than districts in the lower decile, and the districts of Baluchistan depicted extreme spatial heterogeneity in terms of DMI. The findings of the Local Indicator of Spatial Association (LISA) and Moran's test confirmed spatial homogeneity in all mortalities among the districts in Pakistan. The H–H clusters of maternal mortality and DMI were in Baluchistan, and the H–H clusters of child mortality were seen in Punjab. The results of GWR showed that the wealth index quintile has a significant spatial impact on DMI; however, improved sanitation, handwashing practices, and antenatal care adversely influenced DMI scores.

**Conclusion:**

The findings reveal a significant disparity in DMI and spatial relationships among all mortalities in Pakistan's districts. Additionally, socioeconomic, environmental, health, and housing variables have an impact on DMI. Notably, spatial proximity among individuals who are at risk of death occurs in areas with elevated mortality rates. Policymakers may mitigate these mortalities by focusing on vulnerable zones and implementing measures such as raising public awareness, enhancing healthcare services, and improving access to clean drinking water and sanitation facilities.

**Supplementary Information:**

The online version contains supplementary material available at 10.1186/s12889-024-19682-5.

## Background

The post-2015 United Nations Development Program (UNDP) agenda of Sustainable Development Goals (SDGs) is the extension of the Millennium Development Goals (MDGs) that focuses on the commitment to attaining universal health coverage and promotes the principle of inclusivity [1]. As part of the SDGs, the World Health Organization (WHO) advocated for the implementation of Every Newborn Action Plan (ENAP) and Ending Preventable Maternal Mortality (EPMM) targets, which were endorsed by international organizations as well as all member countries [2]. The SDG’s goal 3 mainly aims to “Ensure healthy lives and promote well-being for all at all ages”. In SDG goal 3, target 3.1 specifically aims to decrease the occurrence of maternal mortality rate (MMR), which refers to the death of women during pregnancy or within 42 days following the termination of pregnancy. Simultaneously, Target 3.2 endeavours to eliminate avoidable fatalities of children aged below 5 years, including neonatal mortality rate (NMR), post neonatal mortality rate (PNMR), infant mortality rate (IMR), and child mortality rate (CMR).

Since 1990, progress has been made in the trends of all mortalities worldwide, particularly in low- and middle-income countries [3]. Global child mortalities have decreased from 12 to 5 million, neonatal mortality from 5.2 million to 2.3 million, and maternal mortality ratio by 59% [4]. The world lost 221 million children, teens, and young adults between 2000 and 2022. 162 million of these deaths were children younger than 5 years, 72 million deaths were neonatal, and 91 million were composed of child mortality rate [5]. In addition, the progress in reducing these death rates has come to a standstill after 2010 owing to insufficient investment in maternal, neonatal, and child healthcare [6]. As a result, approximately 6700 newborns and 800 women die every day throughout the globe [2].

Overall, a staggering 5 million children below the age of 5 years have perished with around 2.7 million deaths occurring alone in the year 2021 of which 47% of the deaths were newborns. Among child mortalities, NMR constitutes 50% proportion of total fatalities, which has the lowest progress (from 2000 to 2022) as compared to other U5MRs. Moreover, it is projected that an extra one million maternal fatalities will transpire by 2030, with the majority of these being avoidable [7]. Due to the significance of SDG-3 as a crucial health indicator, it is imperative to conduct a thorough investigation of mortalities particularly in developing countries.

At the midpoint of the SDGs era, developing countries are still off track to achieve the desired targets. For SDGs targets of 2030, 54 countries will be short of achieving the target of child mortalities and 63 other countries will not be able to reach the target of NMR [8]. There are huge geographical variations in maternal and child mortalities around the globe. For instance, the majority of maternal, neonatal, and child deaths have occurred in low-income and lower-middle-income countries [2]. The death probability of a child and maternal mortalities in developing countries is greater by eight times and nineteen times respectively as compared to developed countries [9]. Around 87% of global maternal deaths and 80% of under-five deaths have taken place in Sub-Saharan Africa (SSA) and Asia, respectively.

A child born in a developing country is less likely to survive as compared to a child born in a developed country [10, 11]. In Sub-Saharan Africa (SSA), MMR is 133 times greater as compared to Australia, New Zealand, and Europe. Following SSA, South Asia accounts for 17% of the total maternal deaths worldwide [5]. The prevalence of most maternal and child mortalities in some regions of the globe is indicative of disparities in the availability of high-quality healthcare facilities, hence underscoring the socioeconomic divide between affluent and impoverished populations. Furthermore, the COVID-19 pandemic has aggravated maternal and child mortality worldwide, especially in low- and middle-income countries [12, 13]. Due to such substantial regional differences, the interest of researchers and policymakers has grown in finding spatial disparities in maternal and child mortality, especially in low- and middle-income countries (LMIC).

Pakistan is also facing the challenge of spatial inequality in maternal and child mortality and stands 3rd out of the top 10 fragile countries with the highest maternal and child death burden throughout the globe [2]. Among the South Asian Association for Regional Corporation (SAARC) countries, it has 3rd highest rank in MMR [14] explored that Pakistan's pregnancy outcomes are the worst among the six LMICs. Pakistan is already far away from successfully achieving the targets of MDG related to IMR and MMR. For instance, for Pakistan, the targets of MMR, IMR, and U5MR were 140 per 100,000 live births, 40 per thousand live births, and 45 per thousand live births respectively. Pakistan lags far behind the SDG’s target of 70 deaths per 100,000 live births, 12 deaths per thousand live births, and 25 deaths per thousand live births respectively for MMR, NMR, and CMR [15]. Currently, in Pakistan, the MMR is 154 deaths per 10,000 live births, NMR is about 40 per thousand live births, CMR is 62 per 1000 live births, IMR is 54 per 1000 live births, and U5MR is 65 per thousand live births [16]. Pakistan has a threefold NMR and fourfold IMR as compared to the global target of 2030 [17]. Despite a significant improvement in maternal and child mortalities there exist substantial disparities in health outcomes across the districts of Pakistan [18, 19]. In Pakistan, mortalities are rarely investigated, and no special consideration has been given to assessing the spatial disparities in mortalities. These huge disparities across different geographical boundaries tend the researchers and public health workers to highlight the epidemiology, risk factors, and associated causes that are responsible for differences in mortalities in Pakistan [14, 20–22].

Spatial inequalities in health are intricately linked to the paradigm of social determinants of health (SDH), which explains that the unequal distribution of social, economic, and environmental factors can contribute to differences in health outcomes [23]. The social determinants of health (SDH) encompass indicators such as postnatal care, illiteracy, nutrition, place of delivery, clean drinking water, skilled birth attendance, sanitation facility, and hygiene practices [24]. The mortality rates are different across countries even if they are at the same pace of prosperity due to differences in the functioning of socioeconomic, health, environmental, and household characteristics [25–27]. For instance, 2.2 billion people lack safe drinking water, 3.5 billion people have no access to safely managed sanitation, and 2 billion people lack basic hygiene practices.

In the previous literature much attention has been given to the various socio-economic and health factors associated with maternal and child mortalities, for example, socioeconomic factors such as wealth index, education level, and health factors including distant health facilities, poor health services coverage, antenatal care visits, birth interval, skilled attendance, oral rehydration therapy, and immunization [18, 21, 22, 28–33]. Prior research has discovered that low levels of income and education cause higher maternal and child mortality [34]. However, there are mixed results for the place of residents as [35] documented that the residents of rural areas are more prone to mortalities. In contrast, [36, 37] found that rural areas have a negative association with mortality rates. Similarly, poor health services coverage, antenatal care visits, skilled birth attendance, and postnatal care have positively influenced maternal and child mortality [21, 38, 39]. Besides socioeconomic and health factors, housing and environmental factors also play a crucial role in assessing mortalities. Around 25% of diseases are caused by environmental attributes and children under the age of 15 years are more prone to disease. The environmental effects are more adverse in developing countries as compared to developed countries [40].

In most of the previous studies, an index has been formulated to accurately evaluate health outcomes. These indexes comprised various indicators mainly depending on the core objective(s) of the study. The most important aspect of the index is that developed countries use it to measure their economic growth, particularly their health status [41]. In the realm of health economics, several indices have been estimated to explore the spatial disparities in health outcomes and health services coverage. For example, [42] explored the geographical disparities in social determinants of health using the Urban Health Index (UHI) approach in the City of Atlanta in Georgia (USA) by examining the social determinants using Moran’s I, LISA test, Bayesian correlation tests and spatial error model. They found an improvement in the social determinants of health, and health disparities have decreased in the past 10 years in Atlanta. Similarly, [43] constructed two indices, i.e., socioeconomic development index (SDI) and coverage gap index (CGI) to explore the inequality and coverage gap in maternal and child health services in India. They assessed six health interventions in 642 districts of India and found the spatial clusters among districts by using bivariate and spatial analysis. They also found the difference in coverage gap of CGI in rural and urban areas and poor and rich was clear, which shows huge disparities in service coverage in India.

A similar study has been conducted by [44] to determine the regional disparity in Indonesia by estimating the composite index called the public health development index (PHDI) of health outcomes, health behaviors, public health services, health risks, and infrastructure. They estimated eight indices and found large variations in PHDI scores across different provinces. Western Indonesian provinces have higher PHDI scores as compared to eastern provinces. There are more within-province disparities in the index scores of environment and health services. In addition, [34] measured the spatial effect of determinants of child mortality in Nigeria by estimating the index of maternal mortality and found massive spatial disparities in the contribution of the risk factors on mortality. Wealth index, household head characteristics, maternal education, and toilet facilities were the most effective factors in determining child mortality. The findings also reveal that climate, culture, and political factors affect the chances of child survival. This analysis advocates the importance of spatial evaluation in determining the disparities in determinants of health outcomes through a composite index for a more robust and comprehensive assessment of maternal and child mortality across and within countries [45, 46].

This study is significant based on the following two grounds. First, previous work mainly focused on the measurement of separate (maternal or child) mortalities such as child mortality, infant mortality, neonatal and postnatal mortality [14, 18, 19, 47]. This study combines all (child and maternal) mortality into a composite index to investigate the overall picture of mortality in Pakistan. The composite metric is useful in the measurement and comparison of geographical inequalities in mortality. Besides, it allows the researchers and public health practitioners to choose the size/unit of analysis such as household, union council, district, and province. Second, numerous studies on maternal and child mortality relied mainly on conventional correlation and regression analyses. However, these non-spatial methodologies are incapable of producing spatial outcomes as they do not capture the coordinates information on the study observations. As a result, the coefficient estimates may become biased and inconsistent [21, 36]. Therefore, this study incorporates the spatial technique of Geographically Weighted Regression (GWR) as it covers the coordinates data to find the separate coefficients of the factors associated with maternal and child mortalities for each region and locates the disparities in health outcomes [39, 48].

In a nutshell, previous studies only focused on measuring inequalities in mortalities from non-spatial perspectives, and no proper consideration has been given to investigating the mortalities from spatial/regional perspectives. Considering the significance of mortalities and spatial analyses, the present study is the first attempt to address maternal and child mortalities from spatial perspectives.

Keeping in view the significance of spatial/regional disparities and the SDG’s goal 3 and goal 10, this study has three main objectives. First, it estimates the District Mortality Index (DMI) to measure the inequalities in maternal and child mortality rates across districts in Pakistan. Second, it examines the spatial distribution of maternal and child mortality rates to identify the spatial cluster/randomness among districts of Pakistan as against the descriptive analysis conducted in most of the studies [21, 33, 47, 49]. Thirdly, this study scrutinizes the spatial impact of socioeconomic, environmental, health, and housing determinants on DMI scores. The aim is to furnish valuable insights that can guide policy formulation and interventions. This is crucial for addressing maternal and child mortality in an effective manner and for achieving the pertinent Sustainable Development Goals in Pakistan.

## Methodology

### Area of the study

**Fig. 1:**
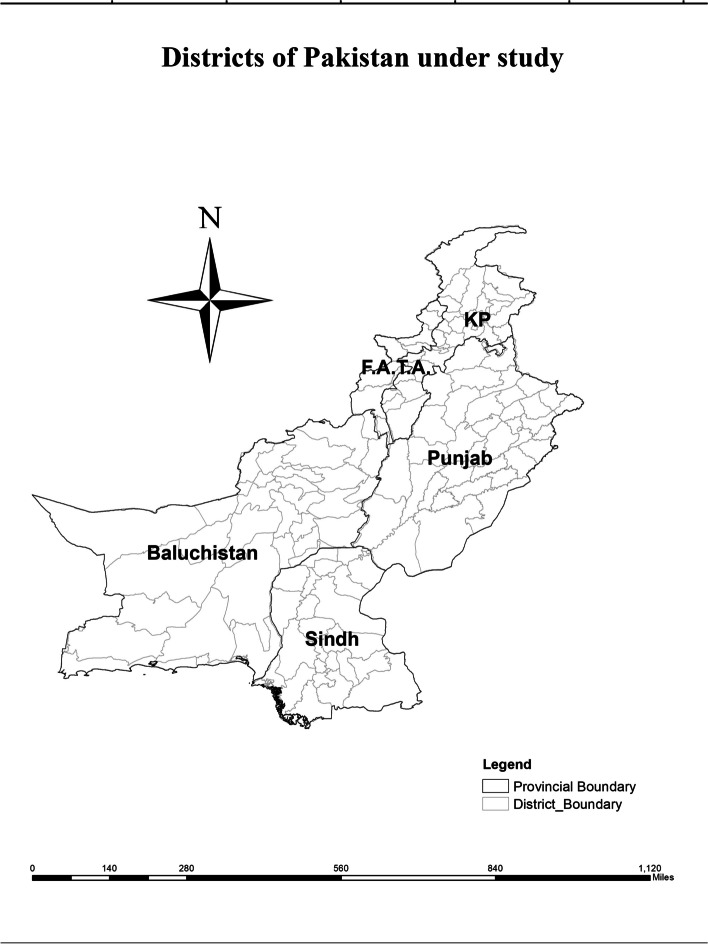
Study area (141 Districts of Pakistan)

This study focuses on the spatial analysis of mortalities across districts in Pakistan's four provinces, namely Sindh, Punjab, Baluchistan and, Khyber Pakhtunkhwa (KP), as well as Federally Administrated Tribal Areas (FATA). Maternal and child mortality rates vary across districts thus susceptible locations may be identified using a spatial analysis of mortalities. As a result, information on mortality statistics for districts is acquired after excluding Azad Kashmir, and northern areas of Pakistan from analysis owing to a lack of essential data. The districts that provide mortality statistics are included as research observations and are illustrated in Fig. 1.

### Data source

This study is based on the micro-level household datasets of Multiple Indicator Cluster Survey(s) (MICS) conducted in 2018–19 by the government of Pakistan in collaboration with the United Nations International Children’s Emergency Fund (UNICEF) [17, 50]. MICS is a nationally representative survey that is segmented by province, including both urban and rural regions, as well as divisions and districts. The data is collected through six questionnaires based on the 2017 Pakistan census of population and housing i.e., women, children ages 5–17 years, children under 5, men, household, and water quality testing questionnaires. It is the most extensive survey composed of a wide range of indicators reporting the health status of children and women i.e., child development, health, nutrition and antennal care, skilled birth attendance, place of delivery, mortality, health services etc. Furthermore, MICS also collects data on key socioeconomic, environmental, housing, and other indicators such as education, income, gender, clean drinking water, sanitation etc.

Maternal and child mortality data is extracted from a questionnaire administered to women and children. Indicators of environmental and household domains are retrieved from household questionnaires, data for health-related indicators are obtained from corresponding questionnaires while socioeconomic indicators are derived from women's and men's questionnaires.

### Sampling design, sample size and unit of analysis

Based on the 2017 Pakistan census of population and housing the sample was selected using a multistage stratified cluster sampling technique [51]. During the initial step, primary sampling units (PSUs) were selected as enumeration areas (EAs), which consist of urban and rural regions within each district. EAs are chosen within each stratum using a systematic method based on their size. The household sample was chosen using a two-stage process; Firstly, the households were enlisted in each EAs and secondly, from the listing of each sample EAs a sample of 20 households was selected through the random systematic procedure to collect data. The PSUs were 5938 EAs and the fieldwork was completed in all except 23 EAs (460 households) in Baluchistan due to covid-19 pandemic.

As the study focuses on geographical variation of maternal and child mortalities across districts and the survey was conducted on the household level the data for the household level has been agglomerated on the district level to execute the study. Therefore, the unit of the analysis is considered a district and all the information from the households were aggregated at the district level. The survey gathered information from 128,040 households and the response rate of the survey was 97%. The sample size was 141 districts in total and Azad Kashmir, northern areas of Pakistan and a few districts from Baluchistan were also excluded due to COVID-19 and deteriorated law and order situation.

### Variables of the study

As this study aims at quantifying the spatial disparities in maternal and child mortalities as well as assessing the impact of socioeconomic, environmental, health and housing factors on DMI, a total of 9 indicators (including dependent variable) have been selected. The outcome variable is “*DMI”* which is a composite of four child mortalities and three maternal mortalities (Table 1).

**Table 1:** Description of various mortalities used in the construction of the District Mortality Index (DMI)

S. No.	Mortality name	Acronyms	Description
1	Neonatal mortality rate	NMR	Probability of dying within the first month of life
2	Post-neonatal mortality rate	PNMR	Difference between infant and neonatal mortality rates
3	Infant mortality rate	IMR	Probability of dying between birth and the first birthday
4	Child mortality rate	CMR	Probability of dying between the first and the fifth birthday
5	Died during pregnancy	DDP	Deaths of women aged 15–49 during pregnancy, excluding accidents and acts of violence, per 100,000 live births
6	Died during childbirth	DDCB	Deaths of women aged 15–49 during childbirth, excluding accidents and acts of violence, per 100,000 live births
7	Died within two months	DW2M	Death of women aged 15–49 within 2 months after pregnancy termination, excluding accidents and acts of violence, per 100,000 live births

Source: Multiple Cluster Indicators Survey (MICS) 2018 and 2019

The exposure variables are *1) “Mother’s Education”, 2) “wealth index quintile”, 3) “Water quality”, 4) “sanitation facility” 5) “Antenatal care”, 6) “Skilled Birth Attendance” 7) “Hand washing”, and 8) “Media exposure”.* These indicators are chosen on different grounds in the context of model specification. Firstly, this study adopts the analytical framework proposed by [27] for indicator selection [27] posit that child mortality rates can diverge between countries with similar economic development levels due to variations in key domains such as healthcare facilities, maternal factors, and environmental conditions. Their model is chosen due to its comprehensiveness, established use in prior research, and particular relevance to the context of developing countries. Also, this model is used interchangeably for studying the determinants of maternal mortality in previous literature [25]. The second strategy relied on examining previous theoretical and empirical research that has shown the impact of the aforementioned factors on mortality rates [25, 27, 52–54]. The third strategy was established based on the health-related issues, requirements, and conditions of maternal and child fatalities in Pakistan. The fourth strategy included the availability as well as accessibility of data, which is the most significant challenge in Pakistan. A comprehensive description of these indicators is provided in Table 2.

**Table 2:** Illustration of domains and associated indicators for measuring DMI

S. No.	Domain	Indicator	Acronym	Explanation	Expected sign	Unit
**Outcome variable**						
1	Index	District mortality index	DMI	A weighted average of all seven mortalities	( -)	
**Exposure variables**						
2	Socioeconomic	Mother’s education	MOED	Percent of mothers aged 15–49 who attended secondary or higher education in a district	( -)	Percentage
3		wealth index quintile	WIQ	Percent of households in the poorest quintile according to the wealth index quintile in the district	( -)	Percentage
4	Environment	Water quality	WQ	Percent of households using improved water sources for drinking in a district	( -)	Percentage
5		Sanitation facility	SANF	Percent of households using improved sanitation facilities in a district	( -)	Percentage
6	Health	Antenatal care	ANC	Percent of the women aged 15–49 with a live birth in the last 2 years who were attended by skilled health personnel in a district	( -)	Percentage
7		Skilled birth attendance	SBA	Percent of the women aged 15–49 with a live birth in the last 2 years who had delivery with the assistance of skilled health personnel in a district	( -)	Percentage
8	Housing	Hand washing	HDW	Percent of households with handwashing facilities where water, soap, or detergent are present in a district	( -)	Percentage
9		Media exposure	MDEX	Percent of women aged 15–49 exposed to any media (watch television, listen to the radio, or read newspaper) at least once a week in a district	( -)	Percentage

Source: Author’s calculations based on Multiple Indicators Cluster Survey (MICS 2018–2019)

Variables 2 to 9 are demonstrated in percentage

### Estimation of district mortality index

DMI is calculated using the procedure proposed by the WHO for evaluating the Urban Health Index (UHI) [46]. UHI was constructed by applying the geometric mean formula as it has two advantages. First, it permits to choice of the number of indicators and the size of the analysis. Second, the single composite metric is beneficial in assessing and comparing spatial inequalities across different localities [55]. This method is also used by [40, 42, 56–58]. DMI is constructed in two steps.

#### Standardization of indicators

To convert the mortality rate into a dimensionless number I^S^ between 0 and 1, the mortality rate was standardized by taking the difference of mortality’s actual value(I) from its lowest value divided by the range of mortality rate [46] denoted in Eq. (2.1) as

2.1 $${I}^{S}= \frac{I- {min}^{*} \left(I\right)}{max\left(I\right)- {min}^{*}\left(I\right)}$$

where ${I}^{S}$ Is the standardized mortality rate and 0 < I^S^ < 1 condition is fulfilled.

#### Accumulation of standardized variables in single matric

In the second step, all standardized indicators have been accumulated into a single metric by taking their geometric mean. Mathematically, the geometric mean is shown in Eq. (2.2) as under,

2.2 $$DMI=\left[\prod_{i=j}^jI_t^s\right]^\frac ij$$

where* j* is the total number of indicators. After assigning equal weights to the mortality rates, a weighted geometric mean was applied to construct the DMI which is indicated in Eq. (3.3) as follows,

2.3 $$DMI=\left[\prod_{i=j}^j{SI}_t^{wi}\right]^{1/\sum\nolimits_{k=0}^nw_{ij}}$$

where *wi* represents the weight of the ith indicator and i = 1,2, 3…,7. Weights were assigned in such a way that their sum equated to the total number of indicators used in DMI.

### Inequality ratio and inequality slope

The inequality ratio is calculated by taking the ratio of the average of the upper 10% of the distribution to the average of the lower 10% of the distribution to identify the disparity gap in better-off and worse-off districts. The inequality slope of the middle 80% of the DMI distribution is obtained using the ordinary least square (OLS) technique (see detailed methodology in Additional File 1).

### Global Moran’s I and local indicators of spatial autocorrelation

In spatial analysis, selecting the appropriate spatial autocorrelation tests is crucial for accurately identifying and interpreting spatial patterns. To examine the spatial relationship in maternal and child mortalities among adjacent districts and identify the location of this spatial association, the present study used Global Moran’s I and local indicators of spatial association (LISA) [22, 36, 56, 59–61]. Moran’s I test is used to comprehend the overall spatial structure of the data and conduct preliminary exploratory spatial data analysis. LISA (Local Indicators of Spatial Association) is employed for in-depth, localized analysis, where the focus is on discovering certain areas exhibiting significant spatial associations. This is particularly useful in heterogeneous landscapes where local variations are crucial. The LISA test also allows for the identification of hotspots, cold spots, and spatial outliers. The combination of these tests ensured a strong and detailed analysis, in line with the research aims and the specific qualities of the data.

Global Moran’s I detects the association between the value of DMI in a district and the mean value of DMI in its adjacent district and aims to test the null hypothesis (H_0_) of spatial heterogeneity against the alternative hypothesis (H_1_) of spatial homogeneity. To measure the extent of the spatial relationship of DMI in an area with contiguous districts, LISA, known as Local Moran’s I, is used. The global and local Moran’s I are expressed in Eqs. (2.4) and (2.5) as follows:

2.4 $$I=\frac{n{\sum }_{i=1}^{n}\sum_{j=1}^{n}{w}_{ij} \left({x}_{i}-\overline{x }\right)\left({x}_{j}-\overline{x }\right)}{{\sum }_{i=1}^{n}\sum_{j=1}^{n}{w}_{ij}\left(x-\overline{x }\right){\left(x- \overline{x }\right)}^{2}} , i \ne j$$

2.5 $${I}_{i}= \frac{n \left({x}_{i}- \overline{x }\right) \sum_{i=1}^{n}{w}_{ij} \left( {x}_{j}-\overline{x }\right)}{\sum_{i=1}^{n}{\left({x}_{i}-\overline{x }\right)}^{2}} , i \ne j$$

where $n$ is the total number of districts, ${w}_{ij}$ is the weighted spatial matrix which shows that if ${w}_{ij}=1$ boundaries of the two districts are adjacent and if ${w}_{ij}=0$ then regions and j are not sharing common boundaries. ${x}_{i}$ and ${x}_{j}$ are the DMI scores for districts i and j. $\overline{x }$ is the mean of DMI scores of all districts and I is Moran statistic. The range of global Moran's I value is from + 1 to -1. A positive value of I indicates that the district exhibits similar DMI scores to its adjacent regions. A negative value of I indicates that a district exhibits different DMI scores compared to its surrounding district. A “0” value means that the DMI scores of the contiguous districts are spread randomly. If the district mortality index value of an area and its neighboring areas is above the mean, then the cluster is a significant cluster of regions with high rates of mortalities.

${I}_{i}$ (in Eq. 5) is the extent of the relationship between I and its adjacent districts. According to [59] the findings of the LISA are categorized into four quadrants: high-high (H–H) or hot spot, low-low (L-L) or cold spot, high-low (H–L) or outlier, and low–high (L–H) or outlier. The H–H and L-L quadrants indicate a high degree of spatial clustering (homogeneity) in the DMI scores of a district and its neighboring districts. On the other hand, the H–L and L–H quadrants represent that a district with high (low) DMI is surrounded by low (high) DMI districts.

### Geographically weighted regression (GWR) model

In the OLS, a single average value of the relationship is obtained i.e. the association between variables is homogenous over space or geographical location. However, the association among indicators may vary across different geographical locations. The conventional OLS technique may inaccurately represent the actual situation, therefore, GWR is considered a more sophisticated model that calculates an average local value of association among indicators and estimates regression parameters for each district [62]. On the contrary, like other econometric models, GWR has several flaws one of them is multi-collinearity in local coefficients. Despite this fact, GWR is considered a useful instrument to detect spatial variation and has been widely used in prior research [19, 48, 63–65].

Conventional OLS determines constant relationships among variables while our interest in this study is to find spatial associations among variables. Therefore, the present study has used the GWR model as it incorporates the role of space in determining the influence of socioeconomic, environmental, health, and housing factors on district composite mortality. Statistically regression model is written in Eq. (2.6) as follows,

2.6 $${Y}_{i}={\beta }_{0}+ \sum_{k=1}^{p}{\beta }_{k} {X}_{ik}+ {\mu }_{i}$$

where Yi represents the exposure/outcome variable at location i = 1,2,3, ––, 141. which is the linear combination of explanatory variables, ${X}_{k}$ at location i, ${\beta }_{0}$ is intercept term and ${\beta }_{k}$ is the regression coefficient of Kth independent variables, k = 1,2,3, 4,…,8, and ${\mu }_{i}$ Is the error term iid independently normally distributed with mean zero and constant variance. OLS is a special case of the GWR model as it does not capture the effect of space or geography in assuming the relationship between exposure and outcome variables. As a result, the true parameters at various topography are captured through the GWR technique. Assuming non-stationarity association among variables GWR measures the district-wise varying coefficients which is a more sophisticated model and can be illustrated in an econometric form in Eq. (2.7) as under,

2.7 $${Y}_{i}={\beta }_{0}\left(ui,vi\right)+ \sum_{k=1}^{p}{\beta }_{k}\left(ui,vi\right){X}_{ik}+ {\mu }_{i}$$

where (ui, vi) shows the coordinates (longitudes and latitudes) of ith location. The district-wise regression parameters are obtained through the application of the GWR method after assigning weights to each unit which is the function of distance from district I. The estimated parameter for each district is depicted in Eq. (2.8) as follows,

2.8 $$\pounds = \left( X^{T}\ W\ (u_{i}, v_{i})\right)^{-1} X^{T}\ W\ (u_{i}, v_{i})Y_{i}$$

where “

$$\pounds$$”

is the vector of unit k at location i = 1,2, 3,..,141. and $\left({X}^{T} W \left({u}_{i}{, v}_{i}\right)\right)$ is a geographically weighted variance covariance matrix. $W \left({u}_{i}{, v}_{i}\right)$ is n × n diagonal matrix of spatial weights with non-zero diagonal elements. (the details of assigning weights are added in additional file 1).

Thus, the Spatial impact of socioeconomic, environmental, health, and housing factors on DMI is examined using the GWR which can be illustrated econometrically in Eq. (2.9) as under,

2.9 $${DMI}_{i}= {\beta }_{0}\left({u}_{i} ,{v}_{i}\right)+ {\beta }_{1}\left({u}_{i} ,{v}_{i}\right) {MOED}_{i}+{\beta }_{2}\left({u}_{i} ,{v}_{i}\right){WIQ}_{i}+{\beta }_{3}\left({u}_{i} ,{v}_{i}\right){SANF}_{i}+{\beta }_{4}\left({u}_{i} ,{v}_{i}\right){HDW}_{i}+{\beta }_{5}\left({u}_{i} ,{v}_{i}\right){ANC}_{i}+{\beta }_{6}\left({u}_{i} ,{v}_{i}\right){SBA}_{i}+{\beta }_{7}\left({u}_{i} ,{v}_{i}\right){WQ}_{i}+{\beta }_{8}\left({u}_{i} ,{v}_{i}\right){MDEX}_{i}+ {\mu }_{0}$$

where $\left({u}_{i} ,{v}_{i}\right)$ are coordinates of the ith district and i = 1,2,3,4,5,….,141.${\beta }_{1}\left({u}_{i} ,{v}_{i}\right)$,${\beta }_{2}\left({u}_{i} ,{v}_{i}\right)$,….,${\beta }_{8}\left({u}_{i} ,{v}_{i}\right)$ are local varying coefficients of the GWR model at location i and ${\mu }_{0}$ is the error term. *DMI* is district mortality index, *MOED* is mother’s education, *WIQ* is wealth index quintile, *SANF* is sanitation facility, *HDW* is handwashing facility, *ANC* is antenatal care visits, *SBA* is skilled birth attendance, *WQ* is household water quality and *MDEX* is mass media exposure in the household.

## Results

### Spatial variations of DMI and associated mortalities in Pakistan

**Fig. 2:**
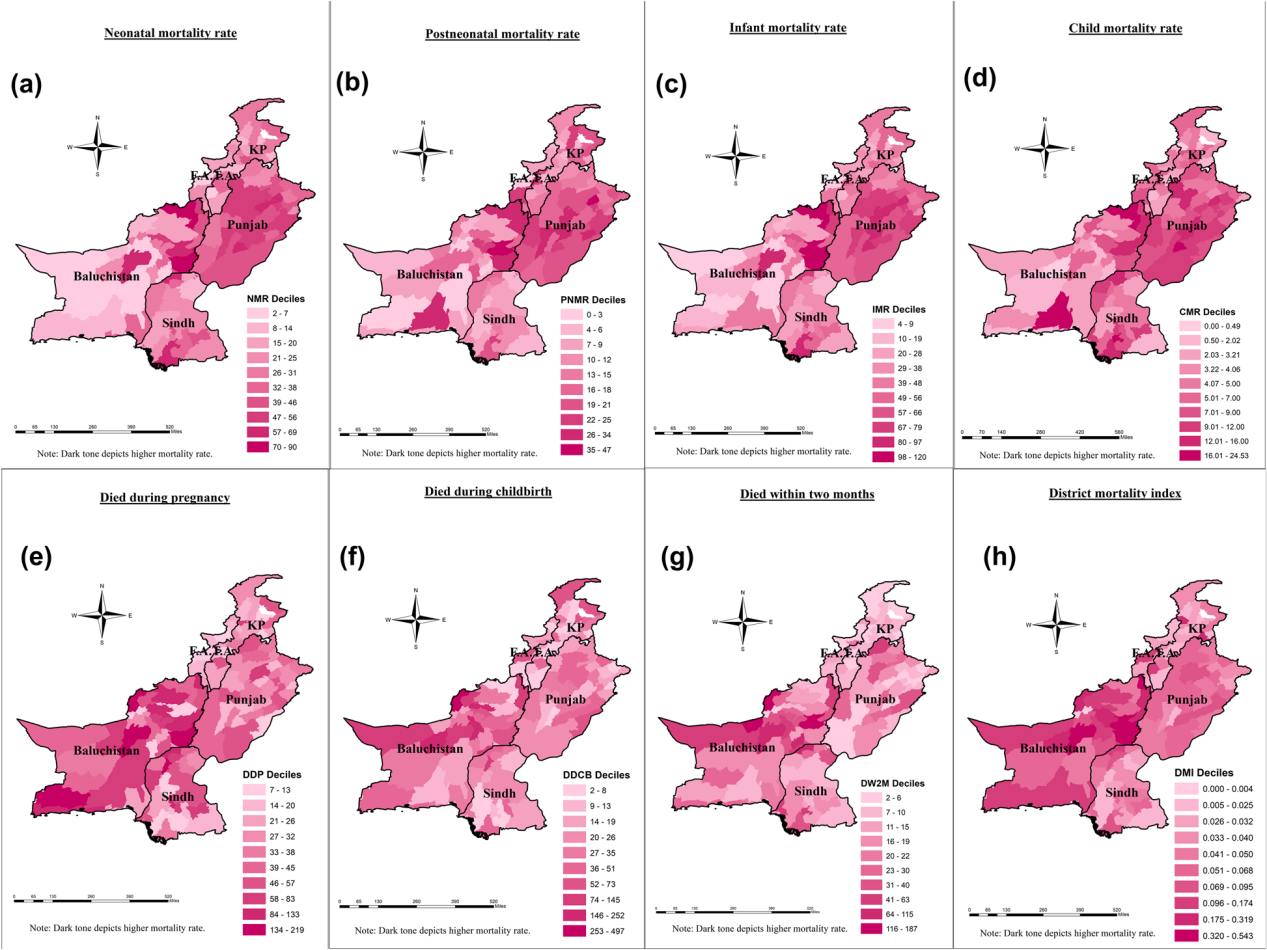
Map for distribution of mortalities and DMI in 142 districts of Pakistan Maps visualize (**a**) neonatal mortality (**b**) Post neonatal mortality (**c**) Infant mortality (**d**) child mortality (**e**) Died during pregnancy (**f**) Died during childbirth birth (**g**) Died within two months after pregnancy termination (**h**) District mortality index. (the map was created by the author using ArcGIS Pro (version 10.8)

Figure 2 displays the visual distribution of child mortalities (panels a to d), maternal mortalities (panels e to g), and district mortality index (panel h). It is shown that child mortalities, including NMR, PNMR, IMR, and U5MR, have a clear spatial pattern of high rates in districts in Punjab province, which means that these mortalities are more severe in Punjab. However, extremely high rates of child mortality were found in Baluchistan province, particularly in the districts of Zhob, Dera Bugti, and Kalat. In contrast to Punjab, spatial clusters of low child mortality were seen mainly in Baluchistan. Both the highest and lowest rates of child (NMR, PNMR, IMR, and CMR) mortalities were clustered in the districts of Baluchistan province, which indicates that the mortality gap is higher in Baluchistan as compared to Punjab, KP, Sindh, and FATA. Moreover, in Sindh, KP, and FATA, these mortalities have made spatial patterns with average rates, except in the southern districts of Sindh, where all mortalities related to children were also spatially bunched with high mortalities. Unlike child mortalities, maternal mortalities (DDP, DDCB, and DW2M) have shown different spatial patterns. All three maternal mortalities with high rates were clustered in districts of Baluchistan province, followed by Punjab (Fig. 2e to g). The highest rates of these maternal mortalities were agglomerated mostly in the western districts of Baluchistan, whereas the lowest rates (concerning DDP, DDCB, and DW2M) were found in different localities from different provinces. For instance, the lowest rates of DDP were spatially clustered in FATA and the western and southern districts of Sindh, whereas concerning DDCB, the lowest rates were seen in Sindh and KP provinces. In terms of DW2M, the southwestern districts of Punjab, KP, and FATA have presented a spatial pattern of low rates. Finally, the district mortality index has presented dramatic results (Fig. 2h). Both Baluchistan and Punjab provinces have displayed clear spatial clusters of high DMI scores; however, these scores were found to be slightly higher in the districts of Baluchistan as compared to Punjab and the rest of the provinces. Interestingly, both the highest and lowest scores of DMI were recorded in the districts of Baluchistan. Such high DMI scores in Baluchistan and Punjab indicate that these areas of mortality are severely stricken. Moreover, Fig. 2g shows that KP, Sindh, and FATA have moderate spatial clusters of district mortality index scores.

The inequality ratio for DMI scores is calculated as "16.93,” which illustrates that the upper decile districts are 16.93 times more prone to mortalities as compared to districts in the lower decile. District "Quetta" has the lowest DMI score, while district "Kohlu" has the highest DMI score, indicating extreme spatial heterogeneity in Baluchistan's districts in terms of DMI. The detailed list of upper and lower decile districts is provided in Additional File 3, Table A. The inequality slope of the midsection (middle 80% of districts) of the distribution was "0.10,” which demonstrates that on average, one unit change in the ranking of a district will change the DMI scores of its adjacent district by 10% (Additional File 3, Table B).

### Hot spot and cold spot zones for mortalities in Pakistan

#### Spatial association and clustering of child mortalities in Pakistan

**Fig. 3:**
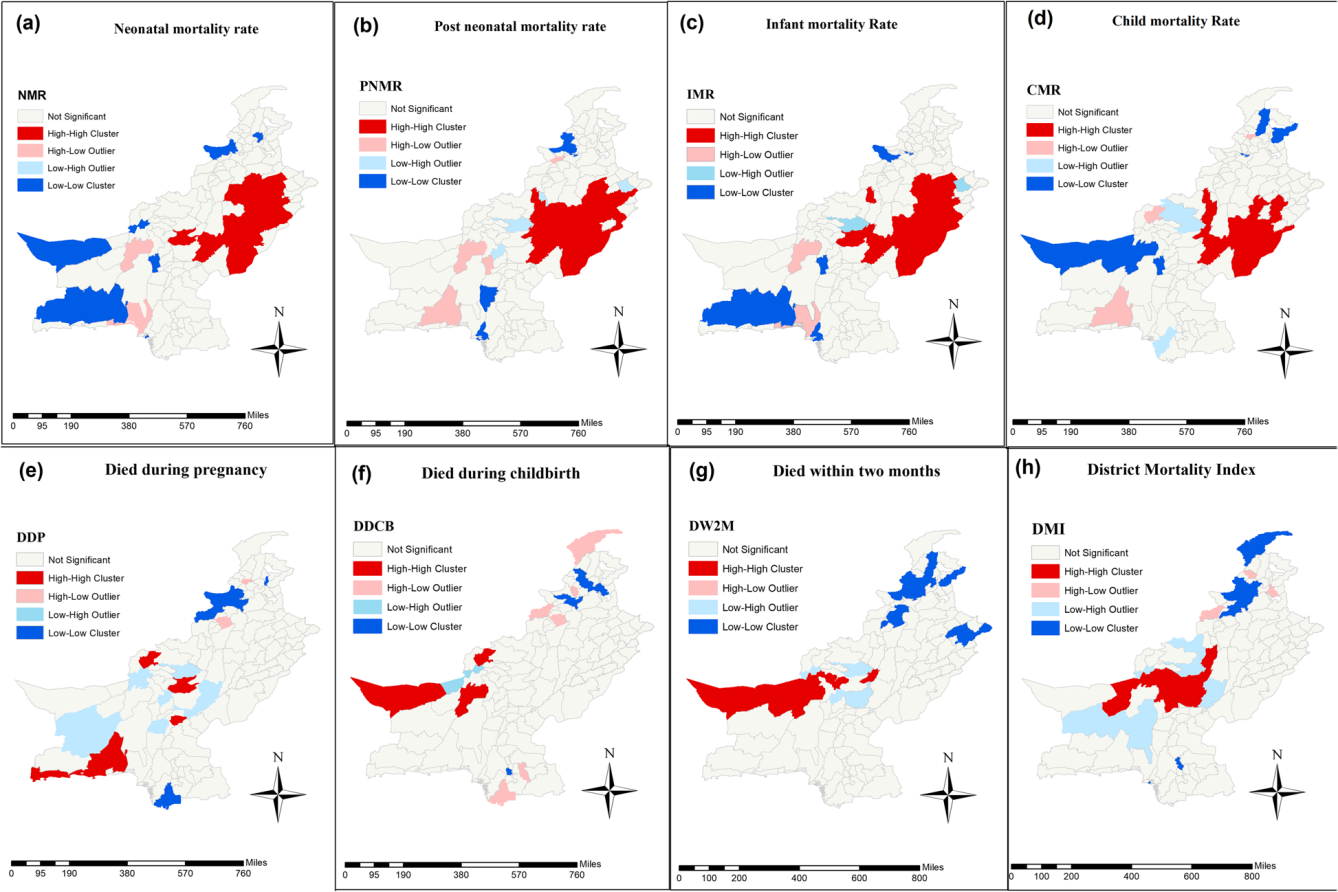
Univariate spatial analysis of LISA test of (**a**) neonatal mortality (**b**) Post neonatal mortality (**c**) Infant mortality (**d**) child mortality (**e**) Died during pregnancy (**f**) Died during childbirth (**g**) Died within two months after pregnancy termination (**h**) District mortality index. The red color represents the H-H cluster and the blue color represents the L-L clusters of mortalities. (these maps were created by the author using the GeoDa application)

The global Moran index value for NMR, PNMR, IMR, and CMR in Table 3 demonstrates a positive spatial autocorrelation among adjacent districts which is around 0.31, 0.30, 0.35, and 0.22 respectively. All the associations are significant (with 999 permutations and pseudo-*p*-value of 0.00) which shows 31%, 30%, 35%, and 22% homogeneity among the mortality rates of the neighboring districts respectively. In order to identify the location of the association among the mortalities, LISA cluster map is drawn (Fig. 3). The H–H clusters of child mortalities were mostly located in central and east-western Punjab and a few districts from eastern Baluchistan whereas the significant L-L clusters were mostly spotted in Baluchistan and lower districts of KP province (Fig. 3a to d). Cold spot (L-L) zones of child mortalities were found to be dispersed. The L-L clusters of NMR were located in south and west Baluchistan, and lower KP areas whereas the significant cold spot zones of PNMR were placed in Sindh and lower districts of KP province. Similarly, the significant IMR clustering in terms of cold spot zones was found in southern Baluchistan few districts from eastern Baluchistan, and lower districts of KP province while the L-L clusters of CMR were found in northwestern Baluchistan and eastern areas of KP province. Moreover, the results also displayed spatial outliers (districts with high (low) mortality rates are surrounded by districts with Low (High) mortality rates) of child mortalities. Most of the spatial outliers (with H–L and L–H characteristics) in child mortalities were situated in Baluchistan and a few districts of Punjab.

**Table 3:** Spatial autocorrelation for all mortalities along DMI measured through Moran’s I

Variable	Moran’s I	E(I)	SD(I)	Z-scores	*P*-values
Neonatal mortality rate	0.316	-0.0071	0.00261	6.325	0.000
Post-neonatal mortality rate	0.301	-0.0071	0.00259	6.056	0.000
Infant mortality rate	0.352	-0.0071	0.00261	7.026	0.000
Child mortality rate	0.226	-0.0071	0.00260	4.580	0.000
Died during pregnancy	0.120	-0.0071	0.00249	2.564	0.010
Died during childbirth	0.073	-0.0071	0.00179	1.894	0.058
Died within two months	0.1887	-0.0071	0.00227	4.109	0.000
District mortality index	0.303	-0.0071	0.00243	6.291	0.000

Source: Authors own calculation based on multiple indicator cluster survey (MICS), 2018–2019

#### Spatial association and clustering of maternal mortalities in Pakistan

The global Moran index for maternal mortality rates in Table 3 indicates a moderately weak correlation among the districts of Pakistan, i.e., the values of Moran’s I for DDP, DDCB, and DW2W were calculated as 12%, 7%, and 19%, respectively. The relationship was found to be significant (with 999 permutations and a pseudo-*p*-value of 0.00) for all maternal mortalities. Moran’s I exhibited spatial homogeneity in the maternal mortality rates among districts of Pakistan. Concerning DDP, the H–H zones were significantly dispersed among districts in Baluchistan, but most of the host spot zones were spotted in southern Baluchistan (Fig. 3e). The highest clusters of DDCB and DW2W were located in north-western Baluchistan and south Baluchistan. The cold spot zones for maternal mortality rates were found in districts of KP province; some significant cold spots for DDP were placed in southern Sindh while concerning DW2W, some substantial cold spot zones are also seen in eastern Punjab. Furthermore, the findings identified spatially significant outliers in maternal mortality. Most of these spatial outliers (with H–L and L–H characteristics) in maternal mortality rates were found in the districts of Baluchistan, as well as in a few districts of KP and Sindh.

#### Spatial association and clustering of DMI

The Global Moran’s I test for DMI scores was measured at 30% (with a pseudo-*p*-value of 0.00 and 999 permutations), denoting a significant association among the mortalities of contiguous districts (Table 3). The LISA analysis of DMI shows that significant hotspot (H–H) areas were located among districts of Baluchistan, especially in southern and central Baluchistan, and cold spot (L-L) zones were spotted in the northern and western districts of KP province (Fig. 3h). The low-low clusters of DMI were also positioned in the central part of Sindh province.

### Diagnostic test of multicollinearity

Before conducting regression analysis to determine the effect of socioeconomic, housing, health, and environmental factors on DMI, it is necessary to examine the severity of collinearity between the variables under consideration. This study examines multicollinearity using the tolerance test and variance inflation factor (VIF). As a baseline, a variable is considered multi-collinear if the value of VIF statistics exceeds 10 and its tolerance level is less than 0.1 [66]. Collinearity poses a substantial issue in regression analysis as it generates deceptive regression results due to a skewed distribution, resulting in inefficient outcomes. The findings of the current investigation indicate that the VIF is less than 10, and the tolerance level surpasses 0.1 for all exposure indicators (Table 4). As a result, the regression findings are statistically significant because there is no severe multicollinearity.

**Table 4:** Collinearity diagnostic test results of study variables

S. no	Name of variable	VIF statistics	Collinearity tolerance
1	Mother’s Education	1.335	0.749
2	wealth index quintile	1.250	0.800
3	Water quality	2.142	0.467
4	Sanitation facility	2.752	0.363
5	Antenatal care	3.572	0.280
6	Skilled Birth Attendance	1.908	0.524
7	Hand washing	1.094	0.914
8	Media exposure	1.566	0.639

Dependent variable: district mortality index

### Error measurement of small area estimation

The estimates from the Empirical Best Linear Unbiased Predictor (EBLUP) model can be used to make more reliable predictions for new observations, especially in areas with limited data. These predictions account for the spatial context and variability, leading to more accurate estimates of the dependent variable. In the present study, the district-level variables were calculated from the MICS data, a considerable amount of data. For a more reliable output of the GWR model from MICS survey data at the district level, the EBLUP model was fitted, which can account for the measurement errors. The performance of EBLUP is evaluated by computing for each small area the Average Relative Bias (AvRBias), the Average Coefficient of Variation (AvCV), leading to less biased estimates of the relationships between variables in the regression analysis, the Average Relative Root MSE (AvRRMSE), and the Average Coverage Rate (AvCR) of nominal 95% confidence intervals [67]. The results of EBLUP are shown in Table 5 show that AvRBias is -0.23%, indicating that the model has a low average predictor error. While AVCV is 21%, demonstrating that EBLUP estimates are precise, the 16% AvRRMSE suggests that the model has a low average prediction error, and the AvCR shows that 73% of the true values fall within the 95% confidence intervals, indicating good interval calibration (details of the error measurement model are given in Additional File 4).

**Table 5:** Summary of results from the EBLUP model

	EBLUP	Min	-1.99036
**AvRBias**	-0.23%	Q_1_	-0.53367
**AvCV**	21%	Median	-0.03583
**AvRRMSE**	16%	Q_3_	0.48886
**AvCR**	73%	Max	3.04658

Source: Author’s calculation

### Geographically weighted regression results

**Fig. 4:**
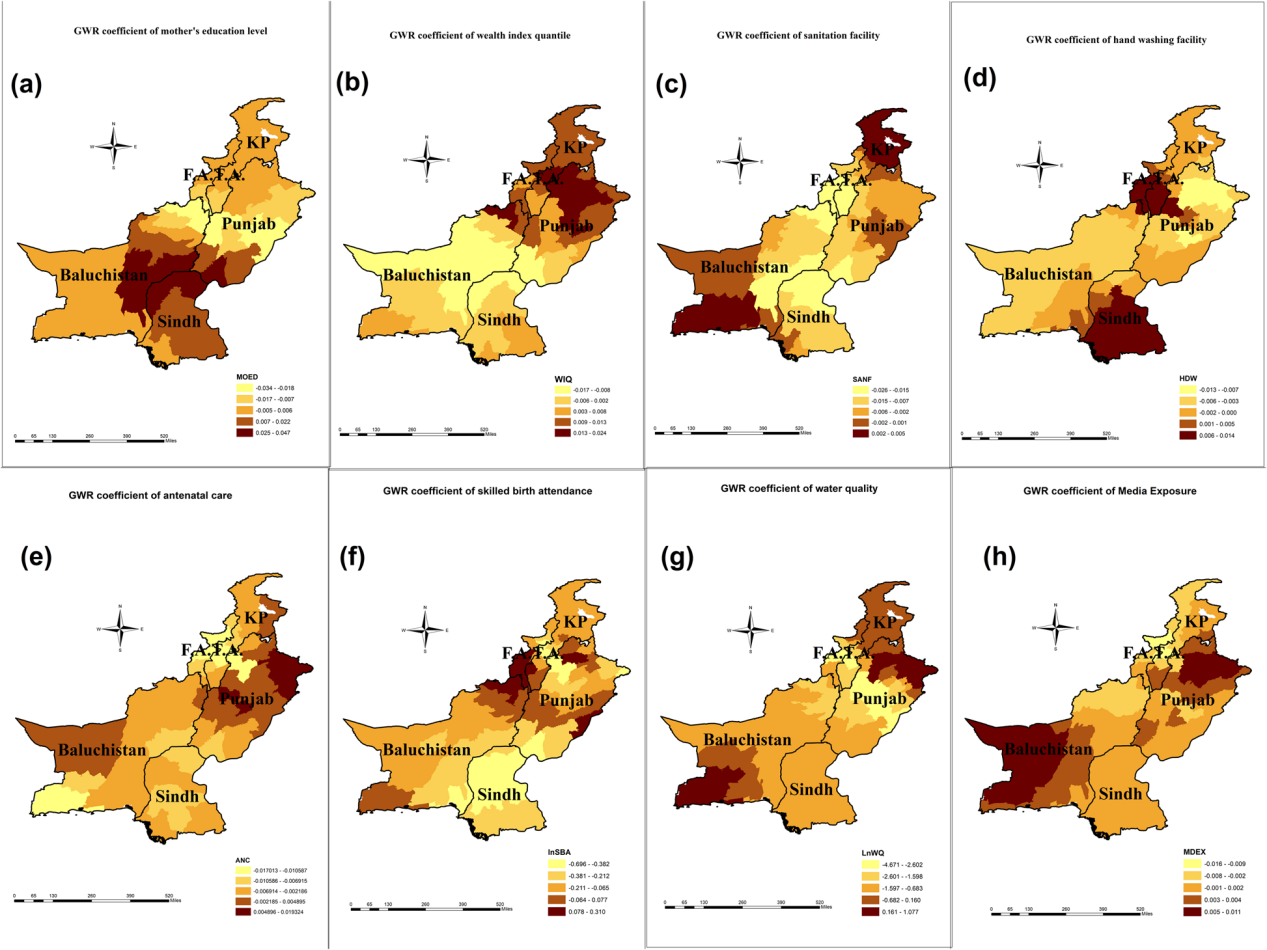
Author's calculations based on Multiple indicator cluster survey(s) (MICS) conducted in 2018–19 in collaboration with United Nations Children Fund (UNICEF) (**a**) GWR coefficient of Mother’s education on DMI score (**b**) GWR coefficient of wealth index quintile on DMI score (**c**) GWR coefficient of sanitation facility on DMI score (**d**) GWR coefficient of handwashing practice on DMI score (**e**) GWR coefficient of antenatal care on DMI score (**f**) GWR coefficient of skilled birth attendance on DMI score (**g**) GWR coefficient of water quality on DMI score (**h**) GWR coefficient of media exposure on DMI score. The dark brown color indicates the highest impact of these indicators on DMI scores. Maps were created by the author using ArcGIS Pro (version 10.8)

The results in Table 6 indicate that GWR yields more robust results as compared to OLS i.e. the values of ML-based global sigma parameters, classic AIC, BIC, Residual Sum of Squares (RSS), -2 log-likelihood, and CV are significantly lowered when local varying coefficients are derived through GWR. Also, when switching from OLS to GWR, R2 improved from 33 to 63%. Under OLS the coefficients of all indicators are negatively associated with DMI except the wealth index quintile which has a significant positive impact on DMI. The locally varying coefficients of the exposure variables in the study for each district are explained through maps in Fig. 4. Source: Author’s calculation based on MICS 2019–2020

**Table 6:** Global (ordinary least square) and local (geographically weighted regression) regression results

**Ordinary Least Square results (Global Regression results)**								
**Variable**	***β *score**	**S.E**	**t-stats**	RSS			60.301	
Intercept	0.004	2.158	0.002	Total number of parameters			9	
MOED	-0.001	0.001	0.034	ML-based global sigma values			0.653	
WIQ	0.010	0.003	2.951^a^	Unbiased global sigma estimates			0.675	
LnWQ	-0.190	0.522	-0.364	CV			0.778	
SANF	-0.006	0.003	-1.781^a^	-2 log-likelihood			280.373	
ANC	-0.013	0.004	-3.245^a^	AICc			302.066	
LnSBA	-0.219	0.197	-1.109	BIC			329.861	
HDW	-0.003	0.001	-3.705^a^	Classic AIC			300.373	
MDEX	-0.002	0.002	-1.010	*R*^2^			0.336	
**Geographically Weighted Regression results (Local regression results)**								
**Variable**	**Mean**	**SD**	**Median**	RSS			33.135	
Intercept	3.024	4.777	3.093	Bandwidth size			58	
MOED	-0.002	0.015	-0.001	ML-based global sigma values			0.484	
WIQ	0.003	0.009	0.006	Unbiased global sigma estimates			0.600	
LnWQ	-1.025	0.970	-1.077	CV			1.137	
SANF	-0.007	0.007	-0.004	-2 log-likelihood			195.949	
ANC	-0.003	0.006	-0.005	AICc			314.035	
LnSBA	-0.181	0.186	-0.154	BIC/MDL			400.438	
HDW	-0.001	0.005	-0.002	Classic AIC			278.591	
MDEX	-0.001	0.004	0.001	*R*^2^			0.63	
				**ANOVA**				
				**Source**	**SS**	**DF**	**MS**	**F**
				Global Residuals	60.30	132		
				GWR improvement	27.16	40.11	0.67	
				GWR residual	33.13	91.88	0.36	1.87

^a^Source: Author's Calculation based on MICS 2018-2019

^a^Shows Significance at 5%

**Fig. 5:**
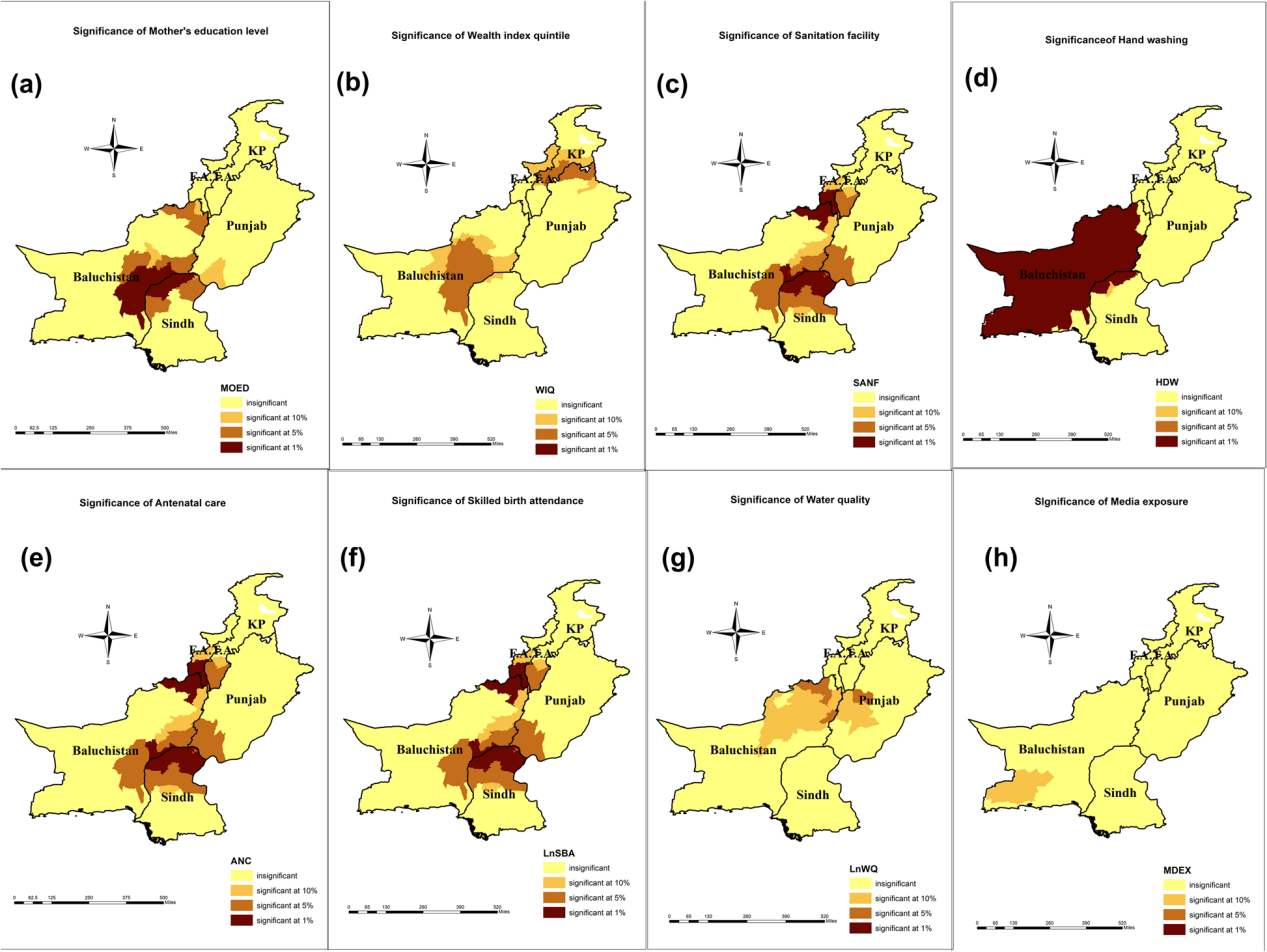
Author's calculations based on multiple indicator cluster survey(s) (MICS) conducted in 2018–19 in collaboration with the United Nations Children Fund (UNICEF). **a** significance level of the mother's education; (**b**) the significance level of the wealth index quintile; (**c**) the Significance level of sanitation facility (**d**) the Significance level of handwashing practice (**e**) the Significance level of antenatal care (**f**) Significance level of skilled birth attendance (**g**) Significance level of water quality (**h**) Significance level of media exposure. The dark brown colour indicates highly significant districts, and the light-yellow colour shows highly insignificant areas. Maps were created by the author using ArcGIS Pro (version 10.8)

The results of local varying coefficients and significant districts are depicted in Figs. 4 and 5, respectively. The analysis indicates a negative association between a mother's education level and DMI, while a positive association is observed with the wealth index quantile (Table 6). It shows that a one percent increase in mothers' education levels will decrease the district mortality index, and the effect size is higher in the central part of Pakistan (Fig. 4a). These districts were located in Baluchistan and Sindh provinces. On the contrary, the effect size was lower in a bunch of districts located in the provinces of Punjab and KP. Figure 5a depicts that the local varying coefficients were highly significant in the southeastern and northeastern districts of Baluchistan and a few districts from northern Sindh.

Similarly, Table 6 displays that a one percent increase in the household population in the poorest quantile will substantially increase the district mortality ceteris paribus. The range of highest impact size (0.013–0.024) was found in northern Punjab, central KP, and a few districts of northeastern Baluchistan (Fig. 4b), and the lowest range was spotted in the central part of the country, especially in districts of Baluchistan. The impact of the wealth index quantile was significant in the districts of eastern Baluchistan, northern Punjab, and central KP (Fig. 5b).

The GWR coefficients of sanitation facilities and hand washing are depicted in Fig. 4c and d, respectively. The empirical findings illustrated that both sanitation facilities and handwashing are negatively associated with the district mortality index (Table 6). The local, varying coefficients of sanitation facilities range from -0.026 to 0.005 (Fig. 4c). The results have shown the highest impact of sanitation facilities on the district mortality index in northern KP and south Baluchistan, whereas a low impact was shown in western Baluchistan and a few districts of eastern Punjab. Figure 5c shows that the local varying coefficients of sanitation facilities were highly significant in northern Baluchistan, southern KP, and northern districts of Sindh province. Handwashing facilities exerted an adverse impact on the district mortality index. An increase in household hand-washing facilities significantly decreased the district mortality index (Fig. 4d). This largest effect was witnessed in Sindh province, a few districts of southwestern KP, and western Punjab. The local, varying coefficients of handwashing facilities were highly insignificant for the districts of Baluchistan and a few districts of Northern Sindh (Fig. 5d).

The GWR results of health factors including antenatal care and skilled birth attendance are presented in Fig. 4e and f, respectively. In Fig. 4e, the highest range of locally varying coefficients of antenatal care was found in the areas of eastern and central Punjab, while a moderately lower range of coefficients was recorded in central Punjab and western Baluchistan, and the lowest coefficients were seen in the rest of the country. Antenatal care was inversely associated with the district mortality index (Table 6). The coefficients in Fig. 5e showed that the impact of antenatal care is highly significant in areas of the FATA region, including districts from northeastern Baluchistan and northern Sindh. Table 6 revealed that skilled birth attendance is directly proportional to the district mortality index, ceteris paribus. Figure 4f indicates that the impact of skilled birth attendance on the district mortality index was highest in upper Baluchistan, lower districts of KP province, and a few districts from Punjab province, while in central Punjab and southwestern districts of Baluchistan, this effect was moderate. Figure 5f shows that the locally varying coefficients of skilled birth attendance were highly significant in areas of the FATA region, districts in northeastern Baluchistan, and northern Sindh.

The GRW results for water quality and media exposure are illustrated in Fig. 4g and h, respectively. Table 6 denotes that water quality was inversely related to the district mortality index. Figure 4g illustrates that the coefficient range (-4.671 to 1.077) and the coefficients for each district have a unique value. Figure 5g shows that water quality was significant in a few districts of Baluchistan and western Punjab. Water quality and media exposure have a negative association with the district mortality index (Table 6). A one percent increase in media exposure will lower the district mortality index, assuming other factors remain constant. Figure 4h shows a cluster of districts with a higher impact, located in western Baluchistan and upper Punjab districts. Locally varying coefficients were found to be significant in two districts of Baluchistan (Fig. 5h).

## Discussion

The global statistics reveal that the world is witnessing maternal mortality every 2 min and under-five mortality every 7 s [2]. SDG targets 3.1 and 3.2 aim to eradicate preventable maternal mortality and mortality among children under the age of five, respectively. Besides, SDG-10 aims to reduce disparities across regions within and between countries [15]. Pakistan is lagging far behind these global targets and has the highest maternal and child mortality among Southeast Asian countries [14]. Consequently, by estimating the district mortality index, the current study quantifies the spatial disparity in maternal and child mortality and identifies the significant influence of several indicators on the district mortality index in Pakistan.

The results of the study revealed extreme spatial heterogeneity in maternal, neonatal, infant, and child mortalities among the districts and four provinces of Pakistan. Similar findings were found in Brazil [68], India [69, 70], 54 African countries [36], and [71], which revealed global subnational inequality in IMR through the Thail index. The visual representation (Fig. 2) depicts that under-5 mortalities were higher in Punjab, while maternal mortality was higher in Baluchistan. Similar findings were also found in the previous studies [22, 47]. Despite being the most developed province in terms of healthcare facilities and literacy, Punjab has the highest child mortality due to its high population density and highest birth rate [72]. Maternal mortality and overall DMI scores were highest in Baluchistan as compared to other provinces, showing heterogeneity in health outcomes across the districts of Pakistan [20]. The highest and lowest district mortality-indexed districts were traced in the province of Baluchistan, which proves Baluchistan is a heterogeneous outlier of DMI. Geographically, Baluchistan is the largest province by area, yet the poorest, deprived, and least developed province [73]. The maternal death surveillance report reveals that most maternal deaths in Baluchistan have occurred due to a low rate of antenatal care visits, distant health facilities, a poor referral system, and delays in referral (resulting from ignorance, poverty, low socioeconomic status, and socio-cultural barriers) [74]. Our study findings also revealed that Sindh and KP are relatively better off in terms of DMI scores, with relatively lower maternal and child mortality rates [75].

The inequality ratio among the districts of Pakistan (16) is greater than the disparity ratio in Sao Paulo (2.5) in Brazil [76]. It depicts that, as compared to cities in Sao Pulao, the upper decile districts in Pakistan with higher DMI scores are more heterogeneous and are less equipped with healthcare resources and opportunities (as compared to districts with lower DMI scores). The disparity slope differs in areas with different geographical locations that are heterogeneous within and among countries. For instance, according to [76], the cities in the census tracts of Sao Pulao are heterogeneous and more sensitive, with a slope coefficient of 0.30 as compared to the slope coefficients in the districts of Pakistan, which are 0.10. As the district's ranking rises, the DMI score increases by 0.10 units (Additional File 3).

The findings of this study provided useful insights into the geographical clustering and randomness of districts in Pakistan concerning all mortalities and the district mortality index. The instant results of global Moran index scores for the child mortality rate demonstrate a significant and positive spatial association, indicating that neighboring districts have similar mortality rates. These results are in line with the findings of [52]. The majority of the districts with spatial associations in neonatal, post-neonatal infant, and child mortality rates were found in central and east-western Punjab and a few districts of eastern Baluchistan. On the contrary, a concentration of distant districts with lower neonatal, post-neonatal, infant, and child mortality rates were located in KP, Sindh, and western Baluchistan. Moreover, the results also displayed spatial outliers (districts with high (low) mortality rates are surrounded by districts with low (high) mortality rates). Punjab is the most developed province of Pakistan and has more healthcare programs as compared to Baluchistan [73]. However, the spatial analysis revealed that Punjab and eastern Baluchistan exhibited higher under-5 mortality rates, with hotspot zones referred to as H–H clusters.

Similarly, the Global Moran index scores of maternal mortality indicators exhibited positive spatial autocorrelation, resulting in spatial homogeneity in maternal mortality among the districts of Pakistan. The results of the LISA showed that most of the contiguous districts with higher maternal mortality rates were located in Baluchistan. These findings were in line with [22], who found LL clusters of maternal health service utilization in Baluchistan. The L-L clusters for maternal mortality were located in KP, southern Sindh, and eastern Punjab districts.

Moreover, concerning overall mortalities (DMI), scores denote significant and moderate association among the mortalities of contiguous districts. The LISA cluster map of DMI showed that H–H clusters of overall mortalities were located among districts of Baluchistan, especially in the districts of central Baluchistan. Similar findings are supported by the study of [49]. The L-L clusters were spotted in the districts of the upper and central districts of KP and the central districts of Sindh province. Baluchistan is the most impoverished region, and a dearth of healthcare facilities could potentially contribute to higher maternal and overall mortality rates.

The GWR results indicated that the mother's educational attainment has a negative spatial effect on DMI scores, albeit to varying degrees across districts [77]. The global review of 52 countries also confirms that each additional year of a mother's education level is associated with a diminution in mortality rates [54, 78]. The instant results confirmed that the districts of Baluchistan and Sindh province were highly sensitive as compared to the districts of KP and Punjab, perhaps due to the low literacy rates in Baluchistan and Sindh as compared to Punjab and KP [79]. The results also confirmed that the wealth index quintile (poorest quintile) had a significant and positive impact on DMI scores. In the impoverished wealth index quintile, there are more fatalities than in the wealthy quintile. This direct association is also in line with previous studies [14, 21, 30, 38, 61, 78]. The highest impact was observed in districts of northern Punjab, central KP, and a few districts of northeastern Baluchistan. Approximately 64% of rural residents fall within the poorest quintile and are unable to afford healthcare services [79, 80]. The locally varying coefficients of the mother’s education level and wealth index quintile have different size effects on DMI scores in each district. Similar findings were found in a study of 72 low- and middle-income countries [53, 75].

The combination of basic sanitation and personal hygiene is widely acknowledged as a crucial factor in ensuring a healthy lifestyle. Inadequate measures to implement a competent sanitation system and the provision of handwashing facilities may expose the community to the peril of an epidemic of contiguous infections [81, 82]. Sanitation facilities and handwashing facilities have a negative association with the district mortality index. Improved sanitation and hygiene practices for hand washing both decrease the chances of maternal and child mortality [32, 83, 84]. The intensity of the effect was different for different districts; for instance, the highest impact of sanitation facilities on the district mortality index was observed in northern KP and southern Baluchistan, while for handwashing facilities, the highest coefficients were witnessed in Sindh province, a few districts of southwestern KP, and western Punjab.

Skilled birth attendance and antenatal care visits are essential in effectively controlling mother and child mortality. Both antenatal care and skilled birth attendance were negatively correlated with the district mortality index, and there was diversity in the intensity of the association among the districts of Pakistan. Similar findings were supported by previous research [18, 22, 29, 61, 85]. The highest range of locally varying coefficients of antenatal care was found in the districts of eastern and central Punjab, while the impact of skilled birth attendance on the district mortality index was highest in the districts of upper Baluchistan, lower districts of KP province, and a few districts from Punjab province. These findings are also supported by [61, 85–87], who have found spatial disparities in SBA and ANC in developing countries. This may be because 93% of women in Pakistan face complications during pregnancy, while 64% of the total population resides in rural areas. The health facilitation centers in these remote areas are fewer in number, with a shortage of trained staff. Also, treatment seeking during pregnancy is lowest (31%) in Baluchistan as compared to Punjab, which is 57% [33, 80]. In these areas, the patients are mostly attended by family members, relatives, and Dai, who cannot handle the sensitivity and complexity of this difficult situation, resulting in maternal and child mortality [79].

Mothers and children are particularly vulnerable to the health risks associated with unimproved water because of their very sensitive immunological, respiratory, and digestive systems. Water quality has an inverse relationship with the district mortality index. Our study results are consistent with the previous studies [83, 84]. The highest impact of water quality on the district mortality index was found in south Baluchistan and northeastern Punjab. While local varying coefficients are highly insignificant throughout the country, except in two Baluchistan districts, perhaps because 94% of Pakistan's population utilizes enhanced sources of drinking water [79]. Similarly, [81, 88] found inequality in access to water and sanitation across districts of India and their effect on maternal and child mortality [89] also found spatial inequalities in water and sanitation in SSA. Similarly, the mass media widely disseminate information on maternal and child healthcare, which improves mother and child survival rates [31]. Media exposure has a negative association with a district mortality index, which is aligned with the studies of [31, 77]. The coefficients of media exposure were different for each district, but a cluster of districts whose impact was higher was located in western Baluchistan and districts of upper Punjab. It may be that 58% of rural women have no access to mass media [90]. These findings are also supported by [34, 70].

## Limitations of the study

Certain limitations are associated with the present study. In its infancy, the capital city of Pakistan (Islamabad) was excluded from the analysis because Islamabad is the most developed city in the country, and we are more interested in finding the disparities in health outcomes that may cause biasedness in the results. Secondly, due to data unavailability, sociocultural factors have been ignored, which exert a substantial influence on rates of maternal, neonatal, post-neonatal, infant, and child mortality. Moreover, within the realm of spatial analysis, numerous indicators can be added to investigate that may influence the DMI. The research may ultimately be disseminated at a micro level, specifically at the level of the Union Council. Nevertheless, due to the unavailability of coordinate data (latitude and longitude) at the Union Council level, the research was carried out at the district level.

## Conclusion and recommendations

This study aimed to estimate the district mortality index and ascertain the role of space in determining the impact of various factors on DMI scores across Pakistan's districts. The independent variables are composed of the mother’s education level, wealth index quintile, household water quality, sanitation facility, hand washing, antenatal care, skilled birth attendance, and mass media exposure as socioeconomic, environmental, health, and housing indicators affecting DMI. To examine the spatial randomness, clusters, or outliers of DMI in each district, univariate Global Moran I and local indicators of spatial association were applied. To assess the spatial impact of the aforementioned indicators on DMI scores, a geographically weighted regression model is used.

Among the four provinces, the districts of Baluchistan displayed extreme spatial heterogeneity in terms of DMI scores, as the highest and lowest DMI scores were recorded in the districts of Baluchistan. The findings of Moran’s test concluded that there is spatial homogeneity in all mortalities among the districts in Pakistan. The H–H clusters of maternal mortality were located in Baluchistan, and the H–H clusters of child mortality were in Punjab. The H–H clusters of DMI were also positioned in Baluchistan. The L-L clusters of maternal and child mortality, along with DMI scores, were located in KP and Sindh. The instant results of GWR concluded that wealth index quintile, improved sanitation, handwashing practice, and antenatal care have a precisely significant impact on DMI scores. The highest impact of the socioeconomic health and housing indicators was found in most of the districts of Baluchistan and a few districts of Punjab. However, the highest impact on mother’s education level and handwashing facilities was found in a few districts of Sindh, while the highest impact on sanitation facilities and wealth index quantiles was also found in districts of KP.

These findings are of high value to policymakers and health financing bodies, as they provide insights into the inequalities in mortalities among the districts of Pakistan. The comprehensive findings of this study advocate for prioritizing Baluchistan (due to the presence of H–H clusters of maternal mortality) and Punjab (due to the presence of H–H clusters of under-5 mortalities), thereby indicating spatial heterogeneity in maternal and child mortality. A significant proportion of maternal deaths in Baluchistan can be attributed to inadequate healthcare facilities, including a shortage of doctors and other medical personnel, as [22] discovered low-low (LL) clusters of maternal health service utilization in Baluchistan. These fatalities could be prevented with timely and appropriate medical care. As a result, we recommend that the government allocate additional funds to improve the utilization and infrastructure of maternal health services in Baluchistan, thereby reducing maternal mortality in the province [91]. In this regard, the Lady Health Worker (LHW) program, one of the largest of its kind in Pakistan [75], could potentially reduce the high rate of maternal mortality if the quality of the program is improved and a proper referral system is implemented. This could maximize the potential of LHWs to alter maternal mortality in Baluchistan.

Policymakers are also directed towards giving more preference to Punjab, as H–H clusters of under 5 mortalities are found in Punjab. To alleviate the child mortality rate, the government may take such steps to overcome poverty, as districts with low wealth index quintiles have more child mortalities. The government can overcome the high child mortality by improving the financial status of people falling in the poorest wealth index quintile, and emphasis should be given to educationally and economically marginalized mothers, particularly those living in remote communities where child healthcare utilization is comparatively low.

The District Mortality Index (DMI) score findings suggest that there is a pressing need for the government to enhance health services and allocate more funding towards Baluchistan due to the higher overall mortality rate. To increase the availability and accessibility of healthcare services in Baluchistan, significant improvements are required. The government could consider initiating awareness programs, such as social media campaigns, to enlighten the populace about the importance of maternal and child health. Baluchistan has the lowest female literacy rate in the country and the absence of a cottage industrial sector results in limited employment opportunities and high poverty levels. The lack of access to clean drinking water is a significant issue, as women travel long distances to fetch water. In terms of healthcare financing, the situation in Baluchistan is dire, with most health expenditures being directed towards tertiary hospitals located in urban centers and cities. This highlights the need for a more equitable distribution of financial resources towards the availability and accessibility of healthcare services in vulnerable zones. The government might also launch awareness programs to educate people about the significance of improved sanitation and handwashing practices in decreasing maternal and child mortality.

### Supplementary Information

Supplementary Material 1.

Supplementary Material 2.

Supplementary Material 3.

Supplementary Material 4.

## Data Availability

The datasets of the Multiple Indicators Cluster Survey (MICS) are publicly available to all users and researchers. Interested researchers can visit the survey through the given link. https://mics.unicef.org/surveys.

## Abbreviations

UNDP: United Nations Development Programme
MDG: Millennium Development Goal
WHO: World Health Organization
ENAP: Every Newborn Action Plan
EPMM: End Preventable Maternal Mortality
SDG: Sustainable Development Goal
SSA: Sub-Saharan Africa
SAARC: South Asian Association For Regional Cooperation
SDH: Social Determinants of health
GWR: Geographically weighted regression
DMI: District mortality index
FATA: Federally Administered Tribal Areas
KP: Khyber Pakhtunkhwa
UNICEF: United Nations International Children’s Emergency Fund
MICS: Multiple Indicators Cluster Survey
PSU: Primary Sampling Units
EA: Enumeration Areas
UHI: Urban Health Index
LISA: Local Indicator of Spatial Association
OLS: Ordinary Least Square
VIF: Variance Inflation Factor
EBLUP: Empirical Best Linear Unbiased Predictor
AvRBias: Average Relative Bias
AvCV: The Average Coefficient of Variation
AvRRMSE: Average Relative Root Mean Squared Error
AvCR: Average Coverage Rate

## References

[1] SDG Target 3.1 Reduce the global maternal mortality ratio to less than 70 per 100 000 live births. Available from: https://www.who.int/data/gho/data/themes/topics/sdg-target-3-1-maternal-mortality.

[2] World Health Organization: Improving maternal and newborn health and survival and reducing stillbirth: progress report 2023: World Health Organization; 2023. Available from: https://www.who.int/publications/i/item/9789240073678.

[3] World Health Organization: Trends in maternal mortality 2000 to 2020: estimates by WHO, UNICEF, UNFPA, World Bank Group and UNDESA/Population Division: executive summary. Geneva: World Health Organization; 2023. Available from: https://www.who.int/publications/i/item/9789240068759.

[4] Newborn Mortality, Key facts. Avaialble from: https://www.who.int/news-room/fact-sheets/detail/levels-and-trends-in-child-mortality-report-2021.

[5] UNICEF, World Health Organization: Levels and Trends Child Mortality-Report 2023: Estimates Developed by the United Nations Inter-agency Group for Child Mortality Estimation. 2024. Geneva: UNICEF, World Health Organization; 2024. Available from: https://data.unicef.org/resources/levels-and-trends-in-child-mortality-2024/.

[6] Goal 3: Ensure healthy lives and promote well-being for all at all ages. Available from: https://www.un.org/sustainabledevelopment/health/.

[7] THE GLOBAL HEALTH OBSERVATORY. Available from: https://www.who.int/data/gho/data/themes/topics/sdg-target-3_2-newborn-and-child-mortality.

[8] UNIGME: Levels & Trends in Child Mortality Estimates developed by the United Nations Inter-agency Group for Child Mortality Estimation; 2023. Available from: https://data.unicef.org/resources/levels-and-trends-in-child-mortality-2024/.

[9] World Health Organization: Health in 2015: from MDGs, millennium development goals to SDGs, sustainable development goals; 2015. Available from: https://www.who.int/data/gho/publications/mdgs-sdgs.

[10] World Health Organization: Protect the promise: 2022 progress report on the every woman every child global strategy for women’s, children’s and adolescents’ health (2016–2030); 2022. Available from: https://www.who.int/publications/i/item/9789240060104.

[11] Ahmed M, Won Y. Cross-national systematic review of neonatal mortality and postnatal newborn care: special focus on Pakistan. Int J Environ Res Public Health. 2017;14(12):1442. 29168764 10.3390/ijerph14121442 PMC5750861

[12] Ujang IRM, Hamidi N, Ab Hamid J, Awang S, Zulkifli NW, Supadi R, Mohamed NE, Sooryanarayana R. The COVID-19 pandemic and disruptions to maternal and child health services in public primary care Malaysia: a retrospective time-series analysis. BMJ Glob Health. 2023;8(11):e013397. 10.1136/bmjgh-2023-013397 PMC10649372 37949498

[13] Moynihan R, Sanders S, Michaleff ZA, Scott AM, Clark J, To EJ, Jones M, Kitchener E, Fox M, Johansson M. Impact of COVID-19 pandemic on utilisation of healthcare services: a systematic review. BMJ Open. 2021;11(3):e045343. 33727273 10.1136/bmjopen-2020-045343 PMC7969768

[14] Aziz A, Saleem S, Nolen TL, Pradhan NA, McClure EM, Jessani S, Garces AL, Hibberd PL, Moore JL, Goudar SS. Why are the Pakistani maternal, fetal and newborn outcomes so poor compared to other low and middle-income countries? Reprod Health. 2020;17:1–12. 33334329 10.1186/s12978-020-01023-5 PMC7745345

[15] United Nations: Transforming our world: the 2030 Agenda for Sustainable Development (New York: United Nations). 2015: A/RES/70/1; 2015. Avaialble from: https://www.sustainabledevelopment.un.org. Accessed 13 Feb 2017.

[16] Economic Adviser’s Wing: Pakistan Economic Survey 2020–21. Islamabad, Pakistan: Government of Pakistan; 2020–21. Available from: https://www.finance.gov.pk/survey_2021.html.

[17] Bureau of Statistics GoP: Multiple Indicators Cluster Survey (MICS); 2021. Available from: https://mics.unicef.org/surveys.

[18] Memon Z, Fridman D, Soofi S, Ahmed W, Muhammad S, Rizvi A, Ahmed I, Wright J, Cousens S, Bhutta ZA. Predictors and disparities in neonatal and under 5 mortality in rural Pakistan: cross sectional analysis. Lancet Reg Health-Southeast Asia. 2023;15:100178. 10.1016/j.lansea.2023.100231 PMC10442969 37614356

[19] Sheikh MR, Khan SU, Ahmed M, Ahmad R, Abbas A, Ullah I. Spatial spillover impact of determinants on child mortality in Pakistan: evidence from Spatial Durbin Model. BMC Public Health. 2023;23(1):1612. 37612693 10.1186/s12889-023-16526-6 PMC10464234

[20] Arif A, Sherani A, Uzma Q, Alam B, Thom E, Abro A, Ehsan N, Mirwani I, Siddiqa A, Sohail U. Maternal and perinatal death surveillance and response in Balochistan, Pakistan-causes & contributory factors of maternal deaths. J Gynecol Obstet. 2022;10(1):1–5. 10.11648/j.jgo.20221001.11

[21] Patel KK, Rai R, Rai AK. Determinants of infant mortality in Pakistan: evidence from Pakistan Demographic and Health Survey 2017–18. J Public Health. 2021;29:693–701. 10.1007/s10389-019-01175-0

[22] Sarwar A. Mapping out regional disparities of reproductive health care services (RHCS) across Pakistan: an exploratory spatial approach. Asia-Pacific J Reg Sci. 2021;5(3):825–49. 10.1007/s41685-021-00207-6

[23] Marmot M, Wilkinson R. Social determinants of health: Oup Oxford; 2005. Available from: https://global.oup.com/academic/product/social-determinants-of-health-9780198565895?cc=my&lang=en&.

[24] World Health Organization: State of inequality: reproductive maternal newborn and child health: interactive visualization of health data: World Health Organization; 2015. Available from: https://www.who.int/data/inequality-monitor/publications/report_2015_rmnch.

[25] McCarthy J, Maine D. A framework for analyzing the determinants of maternal mortality. Stud Fam Plann. 1992;23(1):23–33. 1557792 10.2307/1966825

[26] Kangmennaang J, Elliott SJ. Towards an integrated framework for understanding the links between inequalities and wellbeing of places in low and middle income countries. Soc Sci Med. 2018;213:45–53. 30056326 10.1016/j.socscimed.2018.07.002

[27] Mosley WH, Chen LC. An analythical framework for the study of child survival in developing countries. Bull World Health Organ. 2003;81:140–5. 12756980 PMC2572391

[28] Kumar C, Singh PK, Rai RK. Under-five mortality in high focus states in India: a district level geospatial analysis. PLoS One. 2012;7(5):e37515. 22629412 10.1371/journal.pone.0037515 PMC3356406

[29] Ljungblad LW, Sandvik SO, Lyberg A. The impact of skilled birth attendants trained on newborn resuscitation in Tanzania: a literature review. Int J Africa Nurs Sci. 2019;11:100168.

[30] Barros AJ, Wehrmeister FC, Ferreira LZ, Vidaletti LP, Hosseinpoor AR, Victora CG. Are the poorest poor being left behind? Estimating global inequalities in reproductive, maternal, newborn and child health. BMJ Global Health. 2020;5(1):e002229. 10.1136/bmjgh-2019-002229 PMC7042578 32133180

[31] Fatema K, Lariscy JT. Mass media exposure and maternal healthcare utilization in South Asia. SSM-Population Health. 2020;11:100614. 32596437 10.1016/j.ssmph.2020.100614 PMC7306581

[32] Cameron L, Chase C, Suarez DC. Relationship between water and sanitation and maternal health: evidence from Indonesia. World Dev. 2021;147:105637. 10.1016/j.worlddev.2021.105637

[33] Midhet F, Hanif M, Khalid SN, Khan RS, Ahmad I, Khan SA. Factors associated with maternal health services utilization in Pakistan: evidence from Pakistan maternal mortality survey, 2019. PLoS One. 2023;18(11):e0294225. 37972097 10.1371/journal.pone.0294225 PMC10653445

[34] Gayawan E, Turra CM. Mapping the determinants of child mortality in Nigeria: estimates from mortality index. African Geographic Rev. 2015;34(3):269–93. 10.1080/19376812.2015.1039553

[35] Ezeh OK, Agho KE, Dibley MJ, Hall J, Page AN. Determinants of neonatal mortality in Nigeria: evidence from the 2008 demographic and health survey. BMC Public Health. 2014;14(1):1–10. 24886517 10.1186/1471-2458-14-521 PMC4049428

[36] Yaya S, Anjorin SS, Adedini SA. Disparities in pregnancy-related deaths: spatial and Bayesian network analyses of maternal mortality ratio in 54 African countries. BMJ Glob Health. 2021;6(2):e004233. 33619040 10.1136/bmjgh-2020-004233 PMC7903077

[37] Ogbo FA, Ezeh OK, Awosemo AO, Ifegwu IK, Tan L, Jessa E, Charwe D, Agho KE. Determinants of trends in neonatal, post-neonatal, infant, child and under-five mortalities in Tanzania from 2004 to 2016. BMC Public Health. 2019;19:1–12. 31500599 10.1186/s12889-019-7547-x PMC6734430

[38] Nisar YB, Dibley MJ. Determinants of neonatal mortality in Pakistan: secondary analysis of Pakistan Demographic and Health Survey 2006–07. BMC Public Health. 2014;14:1–12. 24972633 10.1186/1471-2458-14-663 PMC4082298

[39] Gayawan E, Adarabioyo MI, Okewole DM, Fashoto SG, Ukaegbu JC. Geographical variations in infant and child mortality in West Africa: a geo-additive discrete-time survival modelling. Genus. 2016;72:1–20. 10.1186/s41118-016-0009-8

[40] Rothenberg R, Stauber C, Weaver S, Dai D, Prasad A, Kano M. Urban health indicators and indices—current status. BMC Public Health. 2015;15:1–14. 25981640 10.1186/s12889-015-1827-x PMC4491866

[41] Ashraf K, Ng CJ, Teo CH, Goh KL. Population indices measuring health outcomes: a scoping review. J Global Health. 2019;9(1):010101. 10.7189/jogh.09.010405 PMC6344069 30701069

[42] Dai D, Rothenberg R, Luo R, Weaver SR, Stauber CE. Improvement of geographic disparities: amelioration or displacement? J Urban Health. 2017;94(3):417–28. 28417293 10.1007/s11524-017-0151-4 PMC5481217

[43] Panda BK, Kumar G, Awasthi A. District level inequality in reproductive, maternal, neonatal and child health coverage in India. BMC Public Health. 2020;20(1):1–10. 31937270 10.1186/s12889-020-8151-9 PMC6961337

[44] Suparmi, Kusumawardani N, Nambiar D, Trihono, Hosseinpoor AR: Subnational regional inequality in the public health development index in Indonesia. Global Health Action. 2018;11(sup1):41–53. 10.1080/16549716.2018.1500133 PMC7011993 30220248

[45] World Health Organization: The economics of social determinants of health and health inequalities: a resource book, vol. 3700: World Health Organization; 2013. Available from: https://www.who.int/publications/i/item/9789241548625.

[46] Weaver S, Dai D, Stauber CE, Luo R. The urban health index: a handbook for its calculation and use. 2014.

[47] Dawood Z, Majeed N. Assessing neo-natal mortality trends in Pakistan: an insight using equity lens. Arch of Public Health. 2022;80(1):1–10. 34983629 10.1186/s13690-021-00767-1 PMC8725521

[48] Raza O, Mansournia MA, Foroushani AR, Holakouie-Naieni K. Geographically weighted regression analysis: a statistical method to account for spatial heterogeneity. Arch Iran Med. 2019;22(3):155–60. 31029072

[49] Afshan K, Narjis G, Qureshi IZ, Cappello M. Social determinants and causes of child mortality in Pakistan: analysis of national demographic health surveys from 1990 to 2013. J Paediatr Child Health. 2020;56(3):457–72. 31774227 10.1111/jpc.14670

[50] Planning & Development Department GoB. Multiple Indicators Cluster Survey (MICS) 2019–2020. 2022.

[51] Pakistan Bureau of Statistics GoP. Pakistan Bureau of Statistics GoP. Pakistan: Populatin Census; 2017.

[52] Adeyinka DA, Olakunde BO, Muhajarine N. Evidence of health inequity in child survival: spatial and Bayesian network analyses of stillbirth rates in 194 countries. Sci Rep. 2019;9(1):19755. 31875022 10.1038/s41598-019-56326-w PMC6930217

[53] Lohela TJ, Nesbitt RC, Pekkanen J, Gabrysch S. Comparing socioeconomic inequalities between early neonatal mortality and facility delivery: cross-sectional data from 72 low-and middle-income countries. Sci Rep. 2019;9(1):9786. 31278283 10.1038/s41598-019-45148-5 PMC6611781

[54] Balaj M, York HW, Sripada K, Besnier E, Vonen HD, Aravkin A, Friedman J, Griswold M, Jensen MR, Mohammad T. Parental education and inequalities in child mortality: a global systematic review and meta-analysis. Lancet. 2021;398(10300):608–20. 34119000 10.1016/S0140-6736(21)00534-1 PMC8363948

[55] Khan SU, Hussain I. Inequalities in health and health-related indicators: a spatial geographic analysis of Pakistan. BMC Public Health. 2020;20(1):1800. 33243192 10.1186/s12889-020-09870-4 PMC7690118

[56] Bortz M, Kano M, Ramroth H, Barcellos C, Weaver SR, Rothenberg R, Magalhães M. Disaggregating health inequalities within Rio de Janeiro, Brazil, 2002–2010, by applying an urban health inequality index. Cad Saude Publica. 2015;31:107–19. 26648367 10.1590/0102-311X00081214 PMC4727964

[57] Coles E, Kruger E, Anjrini AA, Tennant M. The urban dental index: a method for measuring and mapping dental health disparities across urban areas. J Urban Health. 2017;94(2):211–9. 28168544 10.1007/s11524-016-0131-0 PMC5391330

[58] Khan SU, Hussain I. Impact of safe drinking water and clean fuels on health and wellbeing in Pakistan: a spatial analysis. Groundw Sustain Dev. 2021;15:100677. 10.1016/j.gsd.2021.100677

[59] Anselin L. Local indicators of spatial association—LISA. Geogr Anal. 1995;27(2):93–115. 10.1111/j.1538-4632.1995.tb00338.x

[60] Siqueira TS, Silva JRS, do Rosário Souza M, Leite DCF, Edwards T, Martins-Filho PR, Gurgel RQ, Santos VS. Spatial clusters, social determinants of health, and risk of maternal mortality by COVID-19 in Brazil: a national population-based ecological study. Lancet Reg Health-Americas. 2021;3:100048. 10.1016/j.lana.2021.100076 PMC8432892 34541570

[61] Mishra PS, Sinha D, Kumar P, Srivastava S. Spatial inequalities in skilled birth attendance in India: a spatial-regional model approach. BMC Public Health. 2022;22(1):1–13. 35022008 10.1186/s12889-021-12436-7 PMC8756682

[62] Fotheringham AS, Charlton ME, Brunsdon C. Geographically weighted regression: a natural evolution of the expansion method for spatial data analysis. Environ Plan A. 1998;30(11):1905–27. 10.1068/a301905

[63] Chen VYJ, Deng WS, Yang TC, Matthews SA. Geographically weighted quantile regression (GWQR): an application to US mortality data. Geogr Anal. 2012;44(2):134–50. 25342860 10.1111/j.1538-4632.2012.00841.x PMC4204738

[64] Antczak E, Miszczyńska KM. The self-perceived high level of health quality of Europeans–spatial analysis of determinants. J Appl Econ. 2020;23(1):746–64. 10.1080/15140326.2020.1848308

[65] Muche A, Melaku MS, Amsalu ET, Adane M. Using geographically weighted regression analysis to cluster under-nutrition and its predictors among under-five children in Ethiopia: evidence from demographic and health survey. PLoS One. 2021;16(5):e0248156. 34019545 10.1371/journal.pone.0248156 PMC8139501

[66] O’brien RM. A caution regarding rules of thumb for variance inflation factors. Quality Quantity. 2007;41:673–90. 10.1007/s11135-006-9018-6

[67] Chandra H, Salvati N, Chambers R, Tzavidis N. Small area estimation under spatial nonstationarity. Comput Stat Data Anal. 2012;56(10):2875–88. 10.1016/j.csda.2012.02.006

[68] Sousa A, Hill K, Dal Poz MR. Sub-national assessment of inequality trends in neonatal and child mortality in Brazil. Int J Equity Health. 2010;9:1–10. 20815875 10.1186/1475-9276-9-21 PMC2944212

[69] Singh A, Pathak PK, Chauhan RK, Pan W. Infant and child mortality in India in the last two decades: a geospatial analysis. PLoS One. 2011;6(11):e26856. 22073208 10.1371/journal.pone.0026856 PMC3206872

[70] Mishra PS, Kumar P, Srivastava S. Regional inequality in the Janani Suraksha Yojana coverage in India: a geo-spatial analysis. Int J Equity in Health. 2021;20:1–14. 33413412 10.1186/s12939-020-01366-2 PMC7792199

[71] Sartorius BK, Sartorius K. A new multidimensional population health indicator for policy makers: absolute level, inequality and spatial clustering-an empirical application using global sub-national infant mortality data. Geospat Health. 2014;9(1):7–26. 25545922 10.4081/gh.2014.2

[72] National Institute of Population Studies Pakistan: Pakistan demographic and health survey: ICF international; 2017–18. Available from: https://nips.org.pk/publication/pakistan-demographic-health-survey-pdhs-2017-18-main-report.

[73] Kumar V, Ali BS, Choudry E, Khan S, Baig K, Durrani NUR, Ali SR, Durrani Sr NUR. Quality of Neonatal Care: A Health Facility Assessment in Balochistan Province, Pakistan. Cureus. 2022;14(3):e22745. 10.7759/cureus.22744 PMC8970319 35386481

[74] National Institute of Population Studies II: Pakistan maternal mortality survey. In*.*: National Institute of Population Studies; 2020. Available From: https://microdata.worldbank.org/index.php/catalog/3824.

[75] Bhutta ZA, Hafeez A, Rizvi A, Ali N, Khan A, Ahmad F, Bhutta S, Hazir T, Zaidi A, Jafarey SN. Reproductive, maternal, newborn, and child health in Pakistan: challenges and opportunities. Lancet. 2013;381(9884):2207–18. 23684261 10.1016/S0140-6736(12)61999-0

[76] Lima OACPd, Kruger E, Tennant M. São Paulo urban health index: measuring and mapping health disparities. Rev Bras Epidemiol. 2022;25:e220005. 35293426 10.1590/1980-549720220005

[77] Iqbal S, Maqsood S, Zakar R, Fischer F. Trend analysis of multi-level determinants of maternal and newborn postnatal care utilization in Pakistan from 2006 to 2018: evidence from Pakistan Demographic and Health Surveys. BMC Public Health. 2023;23(1):642. 37016374 10.1186/s12889-023-15286-7 PMC10071715

[78] Jabeen S, Mushtaq K, Samie A, Hassan S. Uncovering the rural–urban gap in determinants of infant mortality in Punjab-Pakistan. Sex Reprod Healthc. 2023;38:100918. 37776801 10.1016/j.srhc.2023.100918

[79] Pakistan Bureau of Statistics: Pakistan Social and Living Standards Measurement Survey 2019–20: District Level Survey; 2021. Available from: https://www.pbs.gov.pk/publication/pakistan-social-and-living-standards-measurement-survey-pslm-2019-20-provincial.

[80] Pakistan Bureau of Statistics, ICF. Pakistan demographic and health survey 2017–18: Key Indicators Report. Islamabad: Pakistan Bureau of Statistics; 2019. Available from: https://microdata.worldbank.org/index.php/catalog/3824.

[81] Cheng JJ, Schuster-Wallace CJ, Watt S, Newbold BK, Mente A. An ecological quantification of the relationships between water, sanitation and infant, child, and maternal mortality. Environ Health. 2012;11:1–8. 22280473 10.1186/1476-069X-11-4 PMC3293047

[82] Hathi P, Haque S, Pant L, Coffey D, Spears D. Place and child health: the interaction of population density and sanitation in developing countries. Demography. 2017;54(1):337–60. 28070855 10.1007/s13524-016-0538-y PMC5306240

[83] Ezeh OK, Agho KE, Dibley MJ, Hall J, Page AN. The impact of water and sanitation on childhood mortality in Nigeria: evidence from demographic and health surveys, 2003–2013. Int J Environ Res Public Health. 2014;11(9):9256–72. 25198687 10.3390/ijerph110909256 PMC4199018

[84] Stauber C, Adams EA, Rothenberg R, Dai D, Luo R, Weaver SR, Prasad A, Kano M, Heath J. Measuring the impact of environment on the health of large cities. Int J Environ Res Public Health. 2018;15(6):1216. 29890750 10.3390/ijerph15061216 PMC6025373

[85] Ruktanonchai CW, Nilsen K, Alegana VA, Bosco C, Ayiko R, Seven Kajeguka AC, Matthews Z, Tatem AJ. Temporal trends in spatial inequalities of maternal and newborn health services among four east African countries, 1999–2015. BMC Public Health. 2018;18:1–13. 10.1186/s12889-018-6241-8 PMC6278077 30514269

[86] Teshale AB, Alem AZ, Yeshaw Y, Kebede SA, Liyew AM, Tesema GA, Agegnehu CD. Exploring spatial variations and factors associated with skilled birth attendant delivery in Ethiopia: geographically weighted regression and multilevel analysis. BMC Public Health. 2020;20:1–19. 32977789 10.1186/s12889-020-09550-3 PMC7519489

[87] Dotse-Gborgbortsi W, Tatem AJ, Alegana V, Utazi CE, Ruktanonchai CW, Wright J. Spatial inequalities in skilled attendance at birth in Ghana: a multilevel analysis integrating health facility databases with household survey data. Tropical Med Int Health. 2020;25(9):1044–54. 10.1111/tmi.13460 PMC7613541 32632981

[88] Ghosh P, Hossain M, Sarkar S. Inequality among social groups in accessing improved drinking water and sanitation in India: a district-level spatial analysis. Prof Geogr. 2023;75(3):361–82. 10.1080/00330124.2022.2124181

[89] Pullan RL, Freeman MC, Gething PW, Brooker SJ. Geographical inequalities in use of improved drinking water supply and sanitation across sub-Saharan Africa: mapping and spatial analysis of cross-sectional survey data. PLoS Med. 2014;11(4):e1001626. 24714528 10.1371/journal.pmed.1001626 PMC3979660

[90] Pakistan Bureau of Statistics & ICF: Pakistan demographic and health survey 2017–18: Key Indicators Report; 2019. Available from: https://microdata.worldbank.org/index.php/catalog/3411.

[91] Kumar V, Ali BS, Choudry E, Khan S, Baig K, Durrani NUR, Ali SR. Quality of Neonatal Care: A Health Facility Assessment in Balochistan Province, Pakistan. Cureus. 2022;14(3):e22744. 10.7759/cureus.22744 PMC8970319 35386481

