# Supplementary material for: Measuring spatial inequalities in maternal and child mortalities in Pakistan: evidence from geographically weighted regression
Source: BMC Public Health. 2024 Aug 16;24:2229. doi: 10.1186/s12889-024-19682-5 (PMC11328511; doi:10.1186/s12889-024-19682-5)
Supplement: Supplementary file 1 — Supplementary Material 1. [file 12889_2024_19682_MOESM1_ESM.docx]

**Additional file 1**

**Inequality Ratio**

The inequality ratio may be expressed mathematically in Eq. (1) as follows:

$$\mathrm{Inequality} Ratio=\frac{Mean of the first decile}{Mean of the last decile}\cdots\cdots\cdots\cdots\cdots\cdots\cdots\cdots(1)$$

**Inequality Slope**

In the first step, the DMI scores of all districts were set in descending order, and then the DMI values were regressed upon ranked variables. Mathematically

$${DMI}_{i}=f \left( {Rank}_{i} \right)\cdots\cdots\cdots\cdots\cdots\cdots\cdots\cdots\cdots\cdots\cdots\cdots\cdots\cdots(2)$$

$Where DMI$_i_ is the index value of “i_th_” district, and “Rank_i_” is the rank of that specific district. “f” denotes the functional relationship between the dependent and independent variables. The equation can econometrically be illustrated as under:

$${DMI}_{i}=\alpha+ \beta{Rank}_{i}+ \mu i \cdots\cdots\cdots\cdots\cdots\cdots\cdots\cdots\cdots\cdots\cdots\cdots(3)$$

In Eq. (3) “β” is the inequality slope, which shows the average DMI heterogeneity between each adjacent district and changes with the ranking of districts. As the number of districts was different in each province, the ranking of districts was rescaled by dividing each district’s rank by the total number of districts in the 8 deciles and regressing the DMI values on the rescaled rank variables.

$${DMI}_{i}=\alpha+ \beta{RescaledRank}_{i}+ \mu i\cdots\cdots\cdots\cdots\cdots\cdots\cdots\cdots(4)$$

The slope estimate through Eq. (4) was used to compare the DMI of different provinces with a bundle of districts.

**Global Moran’s I**

Global Moran’s I is expressed in Eq. (5) as follows:

$$I=\frac{n\sum_{i=1}^{n} \sum_{j=1}^{n} w_{ij} \left( x_{i}-\bar{x} \right)\left( x_{j}-\bar{x} \right)}{\sum_{i=1}^{n} \sum_{j=1}^{n} w_{ij}\left( x-\bar{x} \right)\left( x- \bar{x} \right)^{2}} , i \neq j\cdots\cdots\cdots\cdots\cdots\cdots\cdots\cdots(5)$$

Where $n$ is the total number of districts, $w_{ij}$is the weighted spatial matrix which shows that if $w_{ij}=1$boundaries of the two districts are adjacent and if $w_{ij}=0$ then regions i and j do not share common boundaries. $x_{i}$ and $x_{j}$ are the DMI scores for districts i and j. $\bar{x}$ is the mean of DMI scores of all districts and I is Moran statistic. The range of global Moran's I value is from +1 to -1. A positive value of I indicates that the district exhibits similar DMI scores to its adjacent regions. A negative value of I indicates that a district exhibits different DMI scores compared to its surrounding district. A 0 value means that the DMI scores of the contiguous districts are spread randomly. If the district mortality index value of an area and its neighbouring areas is above the mean, then the cluster is a significant cluster of regions with high rates of mortalities.

**Assigning weights to Geographically weighted regression (GWR) model**

The weights are assigned using the Queen contiguity based on the proximity between location “i” and observations around it. Bandwidth in GWR models may be quantified using several methods (Fotheringham et al., 2009). The fixed Gaussian kernel weighting function was employed in this study as it best fits the model and is illustrated in Eq. (6) under,

$$W_{ij}= exp\left[ - \frac{1}{2}\left( \frac{dij}{b} \right)^{2} \right]\cdots\cdots\cdots\cdots\cdots\cdots\cdots\cdots\cdots\cdots\cdots\cdots(6)$$

The variable $dij$ represents the Euclidean distance between two places i and j in geographical space. The variable b represents the bandwidth, which is the radius of the circle that includes points that are still relevant in determining the model parameters. To determine the best bandwidth, one might minimize a diagnostic measure of model goodness-of-fit, such as the cross-validation (CV) score, which evaluates the accuracy of model predictions, or the Akaike information criterion (AIC). Therefore, to get the coefficient of variation of the bandwidth for a GWR model with a given bandwidth b, one has to minimize the following expression

$$\mathrm{CV}= \sum_{i=1}^{n} \sum_{j=1}^{n} {[y_{i}-\hat{y}_{j\neq i} \left( b \right)]}^{2}\cdots\cdots\cdots\cdots\cdots\cdots\cdots\cdots\left( 7 \right)$$

Here $y_{i}-\hat{y}$ is the difference between the actual and estimated value of the unit.

**Reference**

Fotheringham AS, Brunsdon C, Charlton M: **Geographically weighted regression**. *The Sage handbook of spatial analysis* 2009, **1**:243-254.
