# Supplementary material for: Measuring spatial inequalities in maternal and child mortalities in Pakistan: evidence from geographically weighted regression
Source: BMC Public Health. 2024 Aug 16;24:2229. doi: 10.1186/s12889-024-19682-5 (PMC11328511; doi:10.1186/s12889-024-19682-5)
Supplement: Supplementary file 2 — Supplementary Material 2. [file 12889_2024_19682_MOESM2_ESM.docx]

**Additional File 2**

**Table A. District Mortality Index scores of all study districts of Pakistan**

| **District** | **DMI** | **District** | **DMI** | **District** | **DMI** |
| --- | --- | --- | --- | --- | --- |
| **Federally Administered Tribal Areas (FATA)** | | | | | |
| Bajaur Agency | 0.024658 | N. Waziristan | 0.128628 | S. Waziristan | 0.030886 |
| Kurram Agency | 0.025378 | Orakzai Agency | 0.02559 | Khyber Agency | 0.021403 |
| Mohmand Agency | 0.018747 |  |  |  |  |
| **Baluchistan** | | | | | |
| Awaran | 0.142264 | Kharan | 0.194116 | Panjgur | 0.05558 |
| Barkhan | 0.290358 | Khuzdar | 0.046374 | Pishin | 0.148435 |
| Chagai | 0.165428 | Killa Abdullah | 0.312209 | Quetta | 0.00385 |
| Dera Bugti | 0.448865 | Killa Saifullah | 0.134108 | Sherani | 0.437178 |
| Gawadar | 0.135102 | Kohlu | 0.542615 | Sibi | 0.254479 |
| Harnai | 0.318845 | Lasbela | 0.132882 | Washuk | 0.065459 |
| Jaffarabad | 0.094082 | Loralai | 0.067984 | Zhob | 0.04797 |
| Jhal Magsi | 0.095048 | Mastung | 0.079567 | Ziarat | 0.174124 |
| Kachhi | 0.139709 | Musa Khel | 0.241697 | Lehri | 0.120271 |
| Kalat | 0.490944 | Nasirabad | 0.08686 | Sohbatpur | 0.143771 |
| Kech | 0.103394 | Nushki | 0.118667 |  |  |
| **Khyber Pakhtunkhwa (KP)** | | | | | |
| Abbottabad | 0.018509 | Karak | 0.011525 | Shangla | 0.019343 |
| Bannu | 0.036517 | Kohat | 0.021528 | Swabi | 0.029375 |
| Buner | 0.030823 | Upper Kohistan | 0.047601 | Swat | 0.029559 |
| Batagram | 0.030153 | Lakki Marwat | 0.042117 | Tank | 0.140827 |
| Charsada | 0.019852 | Lower Dir | 0.232591 | Torgher | 0.034555 |
| Chitral | 0.038612 | Malakand | 0.035779 | Upper Dir | 0.01798 |
| D.I.Khan | 0.023169 | Mansehra | 0.04152 | Peshawar | 0.028536 |
| Hangu | 0.033199 | Mardan | 0.018277 | Lower Kohistan | 0.019343 |
| Haripur | 0.177302 | Nowshera | 0.03205 |  |  |
| **Punjab** | | | | | |
| Attock | 0.057137 | Jehlum | 0.050384 | Okara | 0.083032 |
| Bahalwalnagar | 0.041896 | Kasur | 0.061197 | Pakpattan | 0.082398 |
| Bahawalpur | 0.060775 | Khushab | 0.082857 | R Y Khan | 0.048028 |
| Bhakkar | 0.051525 | Lahore | 0.025323 | Rahanpur | 0.045585 |
| Chakwal | 0.054223 | Layyah | 0.05261 | Rawalpindi | 0.045818 |
| Chiniot | 0.048317 | Lodhran | 0.071397 | Sahiwal | 0.04084 |
| D G Khan | 0.064238 | M. Bahauddin | 0.036224 | Sargodha | 0.071186 |
| Faisalabad | 0.046303 | Mianwali | 0.05248 | Sheikhopura | 0.031922 |
| Gujranwala | 0.035711 | Multan | 0.021528 | Sialkot | 0.034126 |
| Gujrat | 0.05698 | Muzaffargarh | 0.036731 | T. T Singh | 0.058434 |
| Hafizabad | 0.058197 | nankana Sahib | 0.066832 | Vehari | 0.072739 |
| Jhang | 0.059442 | Narowal | 0.040218 | Khanewal | 0.065385 |
| **Sindh** | | | | | |
| Badin | 0.025484 | Mirpur Khas | 0.043924 | Sujawal | 0.085907 |
| Dadu | 0.049764 | Nausharo Feroz | 0.020349 | Umerkot | 0.060799 |
| Ghotki | 0.026647 | Sanghar | 0.042042 | Karachi central | 0.054062 |
| Hyderabad | 0.036709 | Shahdad Kot | 0.052151 | Karachi west | 0.032343 |
| Jacobabad | 0.11931 | S. Benazirabad | 0.030192 | Karachi south | 0.057524 |
| Jamshoro | 0.021068 | Shikarpur | 0.073192 | Malir | 0.040668 |
| Kashmore | 0.041457 | Sukkur | 0.022247 | Korangi | 0.054062 |
| Khairpur | 0.037405 | T. A Yar | 0.03524 | Karachi east | 0.032343 |
| Larkana | 0.072324 | T. M Khan | 0.055505 | Thatta | 0.037915 |
| Matiari | 0.058571 | Tharparkar | 0.021071 |  |  |
