# Supplementary material for: Measuring spatial inequalities in maternal and child mortalities in Pakistan: evidence from geographically weighted regression
Source: BMC Public Health. 2024 Aug 16;24:2229. doi: 10.1186/s12889-024-19682-5 (PMC11328511; doi:10.1186/s12889-024-19682-5)
Supplement: Supplementary file 3 — Supplementary Material 3. [file 12889_2024_19682_MOESM3_ESM.docx]

**Additional file 3**

**Table A.** **List of upper and lower decile districts of Pakistan concerning DMI scores**

| **S. No** | **Lower decile** | **DMI** | **Upper decile** | **DMI** |
| --- | --- | --- | --- | --- |
| **1** | QUETTA | 0.00385 | KOHLU | 0.542615 |
| **2** | KARAK | 0.011525 | KALAT | 0.490944 |
| **3** | UPPER DIR | 0.01798 | DERA BUGTI | 0.448865 |
| **4** | MARDAN | 0.018277 | SHEERANI | 0.437178 |
| **5** | ABBOTTABAD | 0.018509 | HARNAI | 0.318845 |
| **6** | MOHMAND AGENCY | 0.018747 | KILLA ABDULLAH | 0.312209 |
| **7** | THATTA | 0.019246 | BARKHAN | 0.290358 |
| **8** | SHANGLA | 0.019343 | SIBI | 0.254479 |
| **9** | CHARSADDA | 0.019852 | MUSA KHEL | 0.241697 |
| **10** | NAUSHAHRO FEROZ | 0.020349 | LOWER DIR | 0.232591 |

**Source:** Author’s computations based on “Multiple Indicator Cluster Survey”, 2018-19

**DMI:** District Mortality Index (standardized) scores

**Table B.** **Regression analysis for disparity slope (Overall Pakistan)**

Dependent variable: DMI scores

| **Variable name** | **Coefficient** | **St: error** | **t-ratio** | **Sig:** |
| --- | --- | --- | --- | --- |
| **Constant** | 0.007198 | 0.002829 | 2.544714 | 0.012323 |
| **Rank** | 0.101936 | 0.00491 | 20.76057 | 0.000507 |
|  | R^2^ = 0.79  R^2^ (Adjusted) = 0.79  F-statistic = 431  Sig. (F) = 0.000507 | | | |

**Source:** Author’s computations based on “Multiple Indicator Cluster Survey”, 2018-19

**DMI:** District Mortality Index (standardized) scores
