# Supplementary material for: Measuring spatial inequalities in maternal and child mortalities in Pakistan: evidence from geographically weighted regression
Source: BMC Public Health. 2024 Aug 16;24:2229. doi: 10.1186/s12889-024-19682-5 (PMC11328511; doi:10.1186/s12889-024-19682-5)
Supplement: Supplementary file 4 — Supplementary Material 4. [file 12889_2024_19682_MOESM4_ESM.docx]

**Additional file 4**

**Error measurement of small area estimation**

Spatial heterogeneity and small area effects can bias the parameter estimates in standard regression models. To reduce the mean squared error of the estimates of population parameters, small-area estimation methods were used to combine the direct estimates of the survey with predictions from a model (Ybara & Lohr., 2008). By borrowing information from neighboring areas, EBLUP can provide more efficient estimates of the regression coefficients. This mainly benefits areas with limited data, leading to more precise estimates with narrower confidence intervals. For Error measurement of small area estimation, the results of EBLUP for fixed effect are demonstrated under

**Table 1: Fixed effect model results**

| **S.no** | **Variable** | ***β* score** | **S.E** | **t-scores** | **Min** | -1.99036 |
| --- | --- | --- | --- | --- | --- | --- |
| 1 | Intercept | 0.2296824 | 2.1440920 | 0.107 | Q_1_ | -0.53367 |
| 2 | MOED | 0.0000507 | 0.0010139 | 0.050 | MED | -0.03583 |
| 3 | WIQ | 0.0096519 | 0.0033411 | 2.889 | Q_3_ | 0.48886 |
| 4 | LnWQ | -0.2308926 | 0.5196896 | -0.444 | MAX | 3.04658 |
| 5 | SANF | -0.0053214 | 0.0031853 | -1.671 | Variance | 0.44492 |
| 6 | ANC | -0.0126344 | 0.0040207 | -3.142 | SD | 0.6670 |
| 7 | LnSBA | -0.2380576 | 0.1985868 | -1.199 | AIC | 321.34 |
| 8 | HDW | -0.0033586 | 0.0009165 | -3.665 | BIC | 409.611 |
| 9 | MDEX | 0.000507 | 0.0017556 | -1.016 | Log-Likelihood | 206.307 |

Source: Author’s calculation

The EBLUP model provides insights into both fixed effects and random effects. The fixed effects results of EBLUP are locally invariant, less consistent, and less reliable than the GWR results. The model with the lower AIC is generally preferred because it indicates a better fit with fewer parameters. In this case, the GWR model has a lower AIC as compared to EBLUP, suggesting it fits the data better than the EBLUP model. Also, the standard deviation of the GWR estimates is consistently smaller than the standard error of the EBLUP estimates, which suggests that the GWR model provides more consistent and possibly more reliable local parameter estimates. On the other hand, the EBLUP standard errors are more prominent, which may indicate less precision in the fixed effects predictions. GWR offers localized coefficients, which can be very informative for understanding spatial heterogeneity; understanding the local variations in relationships is crucial (the main objective of this study). Therefore, the present study used GWR to determine the spatial effect of locally varying coefficients on DMI in Pakistan.
